# Integration of enabling methods for the automated flow preparation of piperazine-2-carboxamide

**DOI:** 10.3762/bjoc.10.56

**Published:** 2014-03-12

**Authors:** Richard J Ingham, Claudio Battilocchio, Joel M Hawkins, Steven V Ley

**Affiliations:** 1 Innovative Technology Centre, Department of Chemistry, University of Cambridge, Lensfield Road, CB2 1EW, Cambridge, UK; 2 Pfizer Worldwide Research and Development, Eastern Point Road, Groton, CT 06340, USA

**Keywords:** automation, flow chemistry, hydration, hydrogenation, sustainable processing

## Abstract

Here we describe the use of a new open-source software package and a Raspberry Pi^®^ computer for the simultaneous control of multiple flow chemistry devices and its application to a machine-assisted, multi-step flow preparation of pyrazine-2-carboxamide – a component of Rifater^®^, used in the treatment of tuberculosis – and its reduced derivative piperazine-2-carboxamide.

## Introduction

Enabling synthesis technologies such as flow chemistry are becoming commonplace in modern laboratories (for recent reviews of flow chemistry in synthesis see [1] and [2]). As more groups start to use this technology, there is an increasing demand to expand the capabilities of laboratory apparatus, in particular for the seamless integration of different types of apparatus from different manufacturers so that they can be used simultaneously and synergistically. Although manufacturers generally provide appropriate control software for use with their particular device ecosystem, these frequently have a limited scope and do not always integrate well with other equipment. For commercial reasons, the control software is rarely provided in a format that can be readily extended by the user to implement control of additional hardware.

Whilst the control of multiple devices and instruments is well-developed and standard practice for multi-step continuous processing on a large scale, these control systems tend to be custom built at a high cost and hence they are not usually appropriate for the research environment. However, even on a laboratory scale the ability to connect and share data between different devices is of critical importance for performing complex processes: recently reported examples include matching downstream flow rates to the concentration profile of a dispersed reaction slug [3], the linking of synthesis to purification apparatus [4] and the development of new work-up technologies [5]. Most manufacturers are willing to share control commands for their products, making these applications possible, but extensive work is often required to coordinate a new process into a smooth and simple operation. This may take the form of a significant programming effort to automate the apparatus, or involve the complex manual timing of events to ensure that the desired operational sequence takes place.

Ideally, we need the ability to create a control algorithm for any new process with minimal up-front effort. Furthermore, control software should be sufficiently flexible such that different items of apparatus can be swapped in and out of the integrated system without having to make significant changes to the automation protocol. Following a review of typical software packages and technologies, we chose to implement a framework for running scripted control algorithms that would allow interfaces for new instruments to be prototyped easily. We hoped that by defining a specification for each class of device (i.e., sensors, pumps, heaters, etc.), these unified interfaces would provide the desired flexibility between devices. In this way, more time can be spent on the engineering and chemistry challenges inherent in the synthesis process, rather than the logistics of control system interfaces.

**Figure 1:**
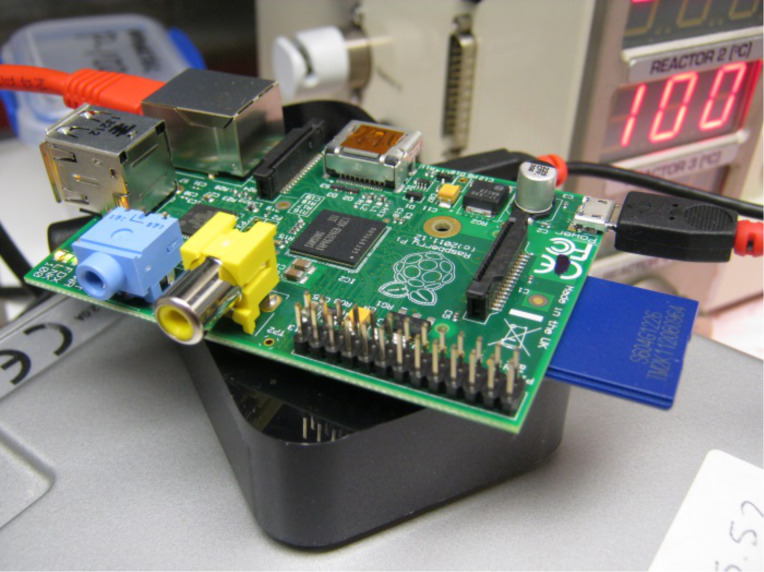
Raspberry Pi^®^ (RPi) computer operating in the laboratory, shown here without its protective case. Underneath is a USB hub (D-Link DUB-H7) which supplies power to the RPi via a short USB cable. The RPi is controlled remotely through the Ethernet connection (red cable) so no monitor, keyboard or mouse is required which reduces the space taken up inside the fume hood. In this case the RPi also communicates with the instruments via the Ethernet connection (the Vapourtec unit pictured in the background is connected using a Brainboxes ES-257 Ethernet-serial converter). Alternatively, a USB-serial connector (such as Lindy P/N 42689) can be plugged directly into the RPi or through the USB hub for serial communication.

Our group has experience using the Python programming language [6] to control laboratory devices [5]. This language claims to be ideal for rapid development and the use of free software fosters collaboration [7], enabling technology to be transferred without the large initial set-up costs typically involved with commercial control packages. Established technologies such as chemical intelligence [8], statistical analysis [9] and computer vision [10–11] are available as third-party libraries for easy integration. The simple text-based control scripts can be copied and pasted for simple re-use, and are compatible with version control systems [12]. Finally, these control programs tend to require low computational resources and will run on cheap, low-power computers such as the Raspberry Pi^®^ (Figure 1) [13].

In the work reported here, we describe the application of automation to performing routine research tasks such as the optimisation of experimental parameters for a particular transformation and describe how the application of remote monitoring can improve safety and efficiency within the research environment.

**Figure 2:**
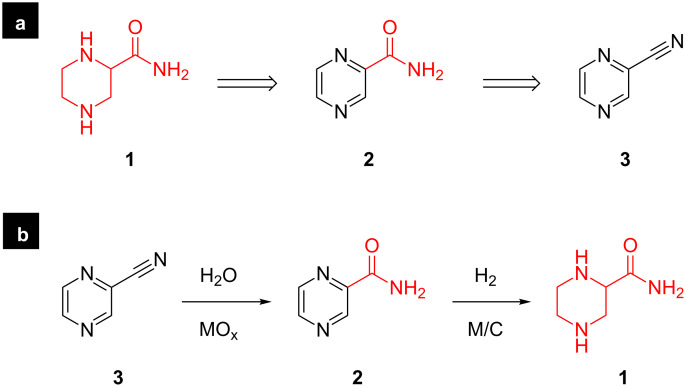
Two step approach to piperazine-2-carboxamide via hydrolysis followed by reduction. (a) Retrosynthesis and (b) catalytic transformations.

In order to test and demonstrate the development of simple inexpensive hardware and software solutions for the facile integration of laboratory hardware, we chose the goal of the efficient synthesis of small molecules which are essential for the generation of fragment-based libraries for medicinal chemistry research programmes. “3D Fragments” have become very attractive recently due to their potential to expand the available chemical space. The presence of nitrogen in a small 3D structure can be important for its biological activity [14]; this is particularly important when developing unnatural amino acid derivatives [15–16]. Such compounds represent an important contribution to a fragment database for medicinal chemistry. For example, piperazine-2-carboxamide (**1**, Figure 2a) is an amino acid derivative with interesting biological properties [17]. At the time of writing, racemic **1** was identified as a notably expensive building block [18] and thus a good target for this transformation. We have explored the possibility of developing a machine-automated synthesis of this compound which might later be extended to analogous structures.

For the facile machine-assisted synthesis of **1** we devised and optimised the fully continuous sequential hydration of nitrile **3** to amide **2** and hydrogenation of pyrazine **2** to piperazine **1** (Figure 2b). Both of these steps involve flowing through heterogeneous catalysts, a metal oxide for the hydration of the nitrile and a supported precious metal for the hydrogenation of the heteroaromatic ring. Furthermore, both steps involve the addition of a volatile small molecule to the substrate with no byproducts: the addition of water for the nitrile hydration, and the addition of hydrogen to the heteroarene for the reduction. Thus, this sequence is ideal for a fully continuous multi-step process.

## Results and Discussion

### Nitrile hydration

**Figure 3:**
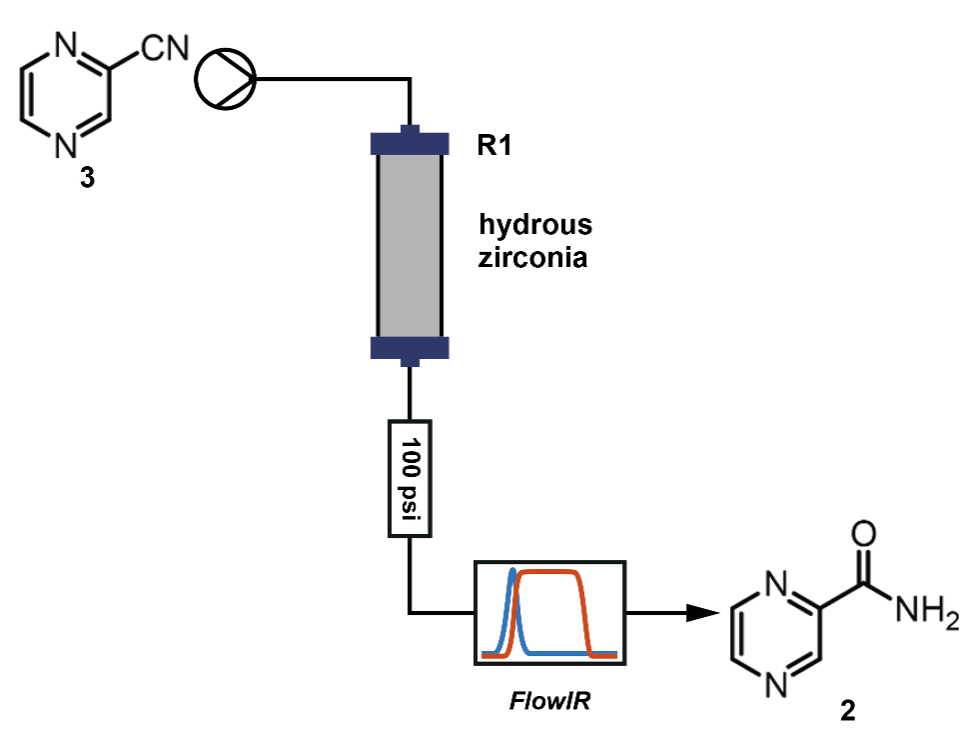
Heterogeneous hydration of pyrazine-2-carbonitrile with hydrous zirconia.

Primary amides can be prepared via a number of different approaches but the most environmentally friendly procedure is the hydration of nitriles [19]. Although this is generally considered to be a simple transformation, there are some inherent problems with the standard techniques of hydrolysis [20]. Further to our previous work which demonstrated the hydration of broad classes of nitriles by passing aqueous–organic solutions through a packed bed of manganese dioxide [21], we have found that heteroaromatic nitriles possessing a β-heteroatom can also be hydrolysed using hydrous zirconia [22–23] in a similar fashion (Figure 3). The directed activity of zirconia is very similar to that of ceria, a known nitrile hydration catalyst for batch reactions [24]. While zirconia has a lower turnover number than ceria, zirconia is less expensive than ceria and it has better physical properties for packed beds than ceria. Its use in packed beds facilitates recycling of the catalyst, providing a further cost advantage.

**Figure 4:**
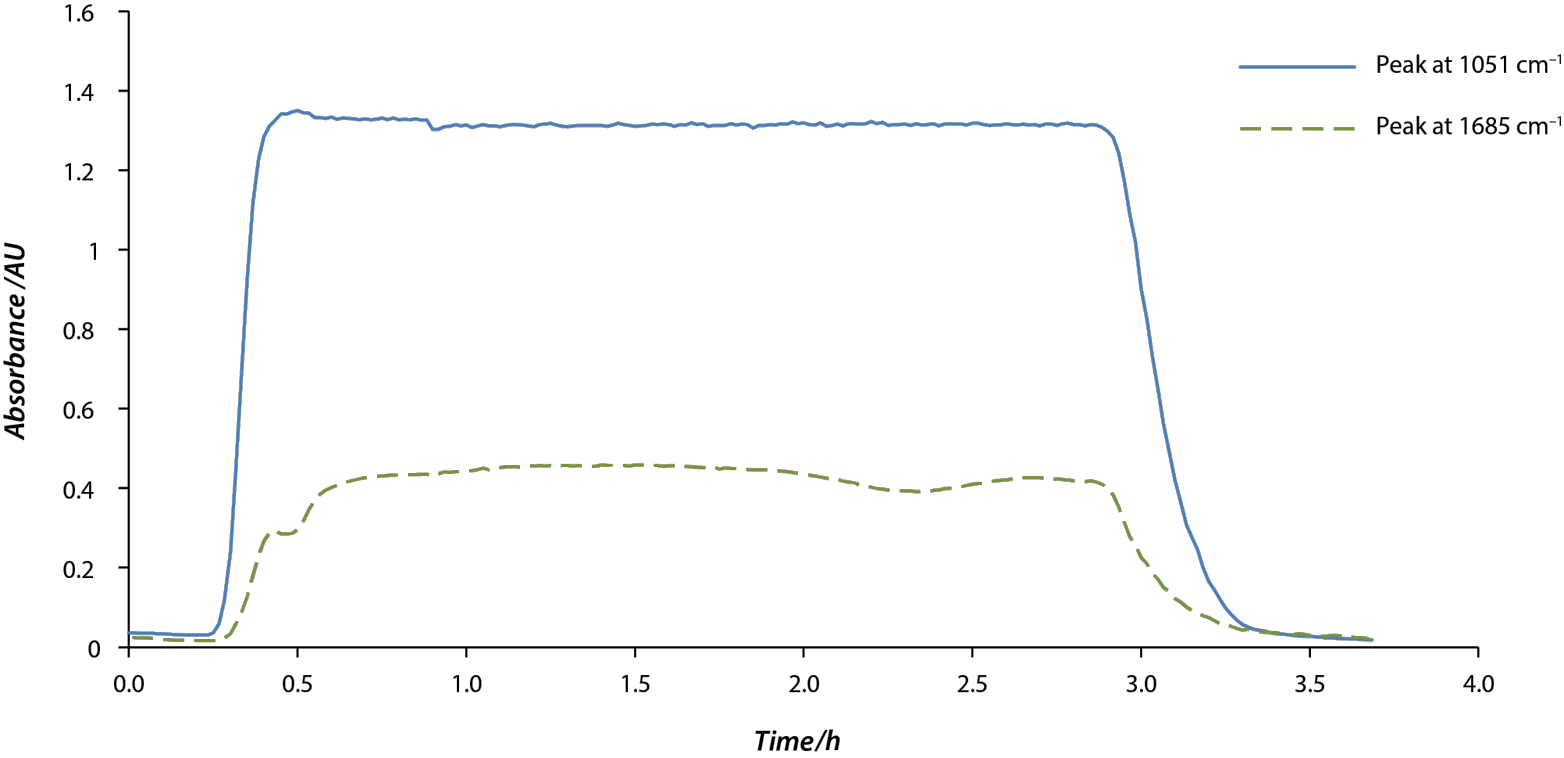
FlowIR™ profile for the reactor output after hydration of pyrazine-2-carbonitrile using hydrous zirconia. Both peaks correspond to the product. A solution of the nitrile in ethanol (0.06 M) was passed at 0.1 mL min^−1^ through the column, heated to 100 °C. The response rises after approximately one column volume, with a small degree of dispersion. It then starts to decrease after the water is used up and the ability of the column to hydrate is exhausted. The fall-off takes place over about one column volume, indicating a low retention of material by the heterogeneous bed.

Interestingly, we have now found that no activation of the zirconia is needed (this is the same as for nitrile hydrations with manganese dioxide, but unlike the use of zirconia as a heterogeneous catalyst for Meerwein–Ponndorf–Verley reductions [22] and Oppenauer oxidations [23] where zirconia activation is required). In fact, it seems that the extent of hydration of pyrazine-2-carbonitrile is proportional to the initial water content of the zirconium catalyst. To confirm this hypothesis, we ran a control experiment in which no additional water was added to the organic solvent (absolute ethanol). A solution of **3** was continuously fed into a reactor column containing untreated zirconium hydroxide [25]. The reaction profile was followed with an in-line infrared (IR) spectrometer in order to determine the conversion. Notably, the hydrolysis process was constant for 3 hours and then the catalytic properties of the system disappeared (Figure 4), suggesting that the surface of the catalyst had been completely dehydrated by the nitrile. As soon as water was added to the reagent solution, quantitative hydration of the nitrile was again achieved for an extended period of time without any further drop in the catalytic activity.

In order to determine the optimal parameters for this reaction, a number of experiments were carried out. The reactor was configured as shown in Figure 3: using a Vapourtec R2+/R4 reactor unit, solutions of nitrile **3** were passed through heated column reactor **R1** (Omnifit^®^ glass column, 100 mm × 6.6 mm; a flow rate of 0.1 mL min^−1^ produced a residence time of 20 minutes) packed with 2.5 g of zirconium hydroxide. (Due to the practicalities involved with performing reactions using different solvents, these tests were run under manual control).

Quantitative transformation to the primary amide **2** was generally achieved within a 20 minute residence time at 100 °C (Table 1). In general, we observed good solvent compatibility; however, when using a water-immiscible solvent such as toluene or ethyl acetate the zirconium hydroxide functioned as a reagent rather than a catalyst. In this case, the reactivity of the metal oxide structure could be regenerated by feeding the reactor with an aqueous solution. As mentioned earlier, it seems to be the water present in the lattice, or fed into the column reactor, which is responsible for the hydration activity of the metal oxide. Consequently, the use of water-miscible solvents is preferred in order to have a continuous process instead of a plug flow protocol using water-immiscible solvents.

**Table 1:** Optimisation for the hydration of the pyrazine-2-carbonitrile. Reactions were performed with dry solvents and a fresh batch of catalyst for each. The reactions were carried out on a 1 mmol plug of pyrazinecarbonitrile such that the zirconia was present in excess.

Solvent^a^	Temperature [°C]	Residence time^b^ [min]	Yield^c^

isopropanol	100	10	49%
isopropanol	100	20	98%
ethanol	100	10	51%
ethanol	100	20	100%
methanol	100	20	100%
ethyl acetate	100	20	45%
toluene	100	20	92%
water	100	20	88%
tetrahydrofuran	100	20	95%
dioxane	100	20	94%

^a^No water was added to the organic solution; ^b^residence time within the column reactor; ^c^the yield refers to that of the isolated product.

Under the optimised conditions, a solution of nitrile **3** in ethanol/H_2_O (0.6 M, 8:1 v/v) was passed through the column reactor **R1** heated at 100 °C, with a residence time of 20 minutes, to obtain a quantitative yield of the primary amide **2** after concentration of the reactor output.

To assist with the processing of a large amount of material, we applied an automated control and monitoring system being developed within our group [26], which can carry out a programmed sequence of operations written using the Python™ language. There is also a remote interface for observing the status of an ongoing reaction in real-time. In common with the industrial use of process analytical technologies (PAT), a number of parameters are read from devices or sensors, and the effluent is only collected when all of the parameters are stable, ensuring a high degree of purity in the collected material.

**Figure 5:**
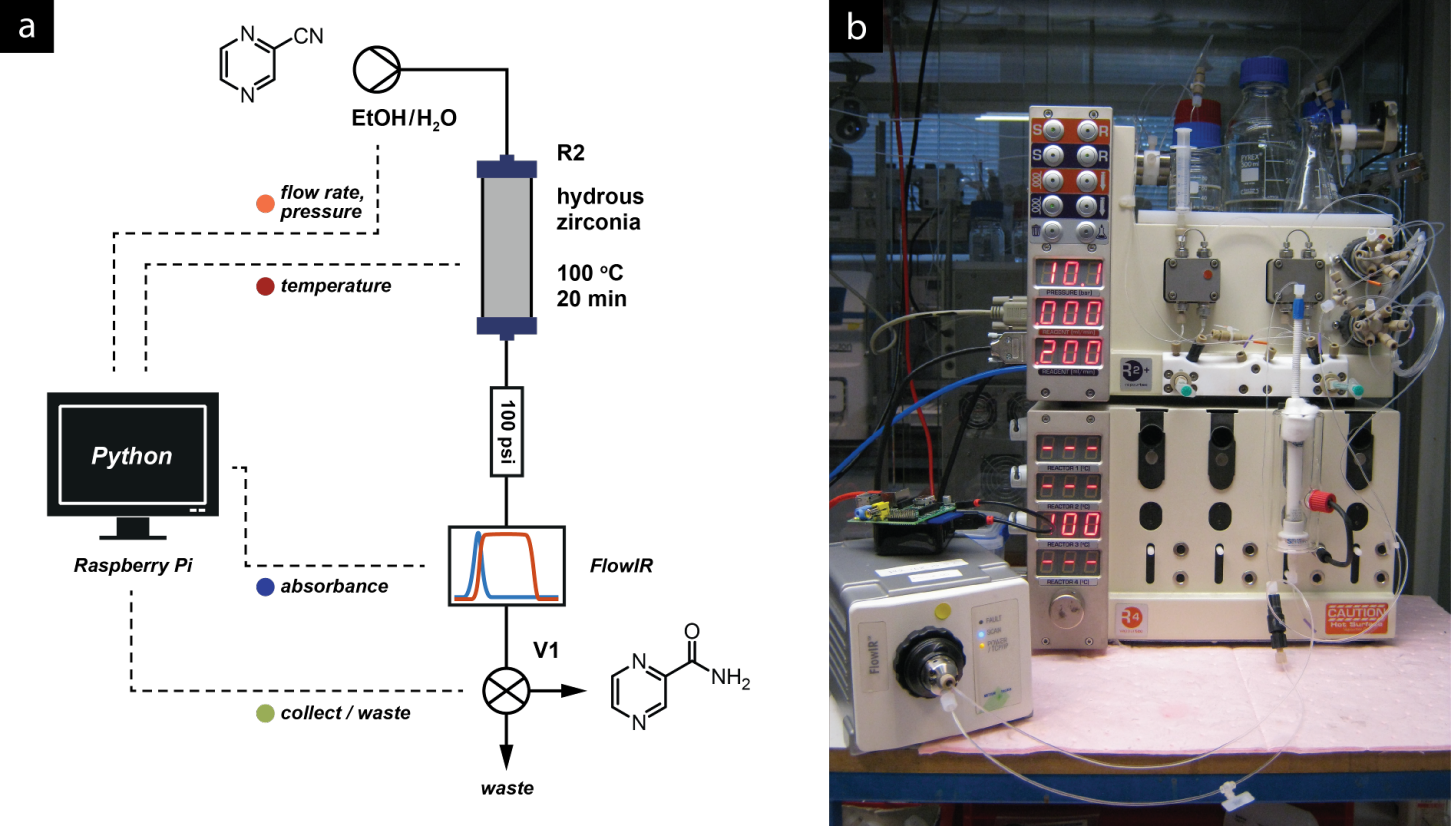
(a) Fluidic setup for the zirconia catalysed hydration of aromatic nitrile. (b) Raspberry Pi^®^ microcomputer, FlowIR spectrometer and Vapourtec R2+/R4 reactor unit as used for this procedure.

Larger zirconia columns were also used to improve the throughput. Employing 5 g of zirconium hydroxide within a 100 mm × 10 mm diameter Omnifit^®^ column (**R2**, Figure 5a) gave a two-fold increase in throughput. Under these conditions, we could use a flow rate of 0.2 mL min^−1^ and still generate a quantitative hydration of the nitrile, generating an output equating to 0.45 g h^−1^ of amide product.

Using a Mettler–Toledo FlowIR™ fitted with a detector with a silicon window (required for visualising the nitrile region of the spectrum, which is blocked by the standard diamond window) the change from nitrile to amide can be observed, providing real-time feedback on the state of the reaction.

**Figure 6:**
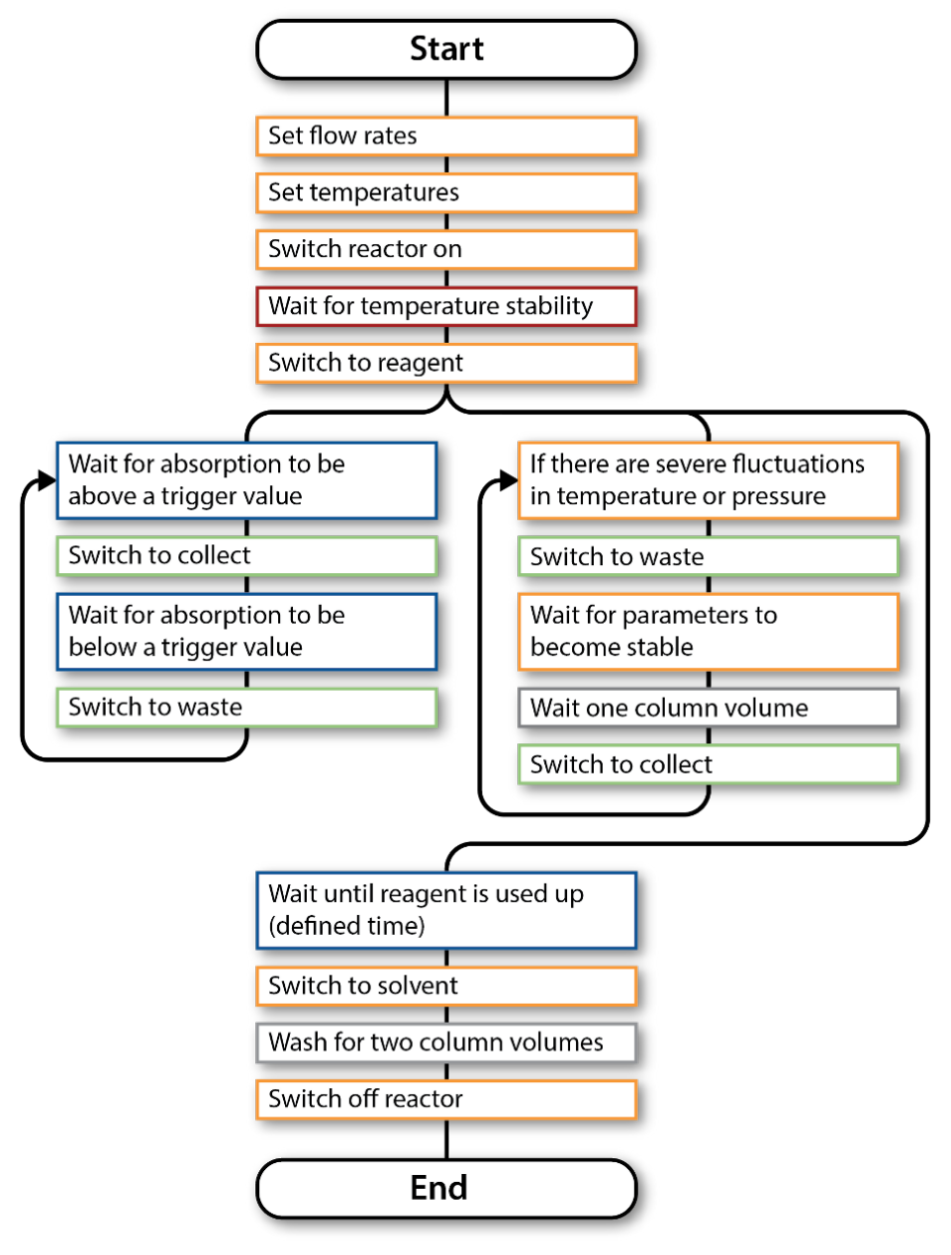
Flowchart describing the control sequence for operating and monitoring the hydration reaction. The black line indicates the execution sequence from start to end. Branch points indicate parallel execution. (See Supporting Information File 1 for the control program).

The monitoring software was connected to the Vapourtec R2+/R4 reactor via RS-232, and to the FlowIR™ spectrometer via the Auto-Export feature of the Mettler–Toledo iC IR control software running on a separate computer. The desired steady-state parameters were defined, and an output valve (**V1**) was controlled based on their states. Importantly, different responses were defined for each parameter: the IR absorbance represents the current reactor output and thus can command immediate responses from the valve. On the other hand, fluctuations in temperature or pressure could compromise an entire column volume, so a delay was added between the time that these parameters stabilised and the time that collection resumed (Figure 6). An alerting system was also included that could notify the operator if the reactor lost pressure, indicating an air bubble or a leak, or if it over-pressured – situations that currently require manual intervention to rectify.

All of the reaction parameters could be observed on remote computers, or wirelessly on a tablet computer when moving around the lab. The monitoring software generates an interface for each running experiment, which can be accessed through a web browser. The ability to access real-time experimental information from anywhere – as opposed to only on computers situated next to the apparatus – is very important, because it gives the chemist freedom to perform other tasks at the same time. This is particularly beneficial when data from multiple reactors and devices is combined into a single interface.

The sensors were interrogated approximately once a second; a Raspberry Pi^®^ microcomputer (as shown in Figure 5b) has more than sufficient processing power to perform the required data collection, interpretation and control. We anticipate that much more complex systems than this one could be controlled using this miniature computer system.

**Figure 7:**
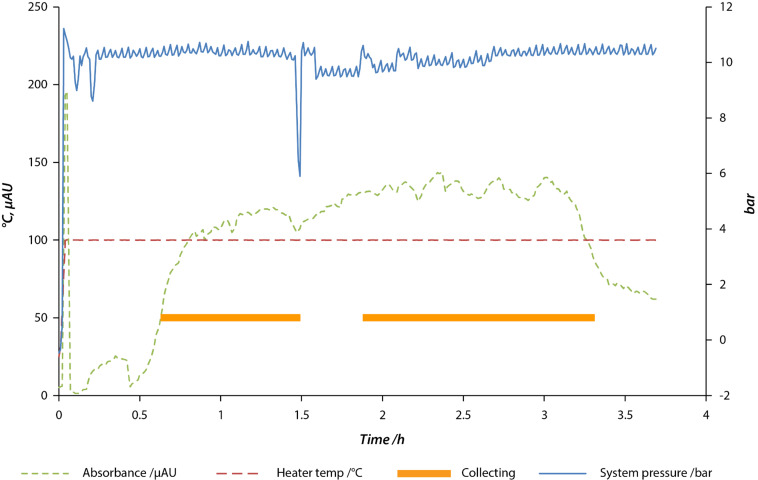
Profile for a 3 hour reaction simulating a long run. The absorbance shown is that at 1685 cm^−1^, which is indicative of the amide bond in **2**. The nitrile stretching absorbance at 2245 cm^−1^ was not observed. The thick bar represents the time over which valve **V1** is set to collect the output as opposed to directing it to waste. The pressure drop at 1.5 h, caused by a bubble in the inlet stream, triggered the system to send the effluent to waste until a predetermined wait time – corresponding to one column volume – elapsed after the pump had been manually re-primed.

An example of the error handling behaviour is shown in Figure 7. After approximately 40 minutes the product begins to elute and after it passes a threshold in the absorbance as detected by the FlowIR™ unit, valve **V1** is switched to collect the output. After approximately 1.5 h, a loss in pressure is detected corresponding to an air bubble in the input stream. Valve **V1** is switched to waste, and an SMS notification is sent to the operator, who re-primes the pump. After the pressure has returned to normal there is a delay calculated to be the dead volume of the column before the output is collected again.

Pleasingly, experiments on scale (50 mmol) gave very positive results. The catalystic activity of the system remained constant and the product **2** was recovered quantitatively and, more importantly, characterised by high purity as determined by elemental analysis (>98%).

### Pyrazine ring reduction

An initial investigation showed that this aromatic carboxamide could be efficiently reduced using an H-Cube^®^ reactor [27–28]. There has been recent interest in the use of automation to optimise reaction conditions [29–31], and thus we hoped to use a linear programming method [32–33] in a similar way to iteratively improve the hydrogenation settings. This turned out to be impractical for two reasons: the flow rate, dead volume, and stabilisation time required for the H-Cube to reach steady state meant that each iteration would take 30–60 minutes; and the discrete nature of the available parameters (for example, the column temperature can only be set in units of 10 °C) mean that simple linear optimisation methods are not suitable [34]. Therefore, we decided to use a Design of Experiments [35–38] method to determine which of the available parameters were important for the conversion and selectivity of this reaction. This process requires a lot of repetitive work to be done, and thus can greatly benefit from reaction automation.

A two-level factorial design with three parameters (temperature: 40 °C and 100 °C; H_2_ pressure: 20 bar and full hydrogen mode; and flow rate: 0.1 mL min^−1^ and 0.2 mL min^−1^) suggested 16 experiments – two repeats each of eight sets of conditions. A single catalyst was used, a 10% Pd/C cartridge supplied by ThalesNano. With a large amount of material from the previous step in hand, we decided to use an automated system to perform these experiments in order to reduce the amount of operator’s time that is required.

**Figure 8:**
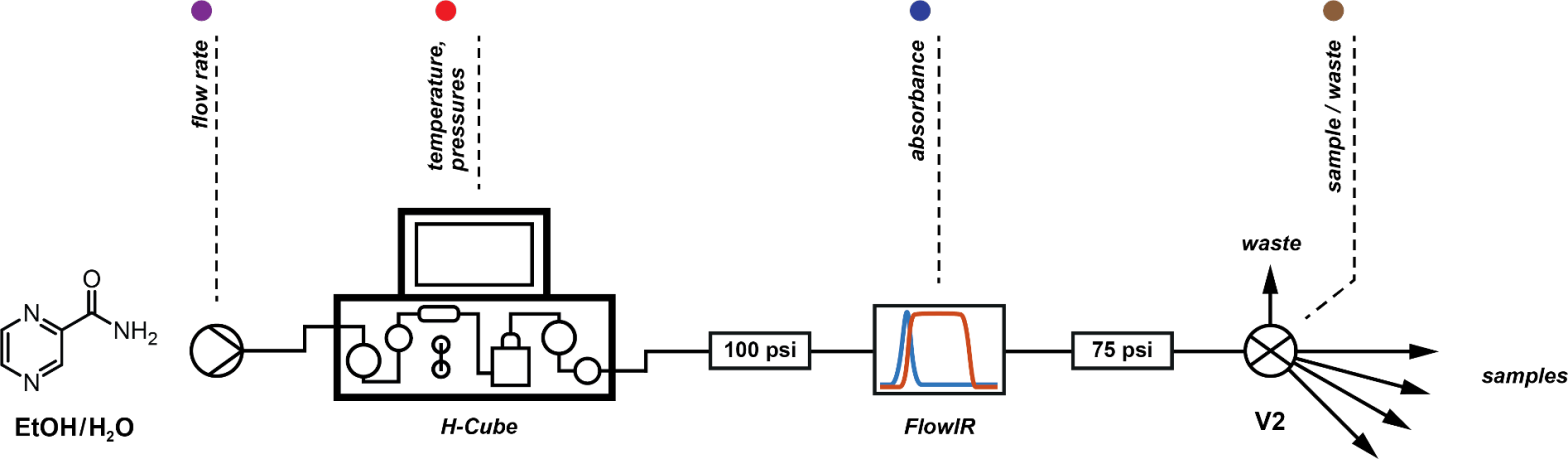
Reactor setup for optimisation reactions. A multi-position valve (**V2**) was used for collecting samples.

**Figure 9:**
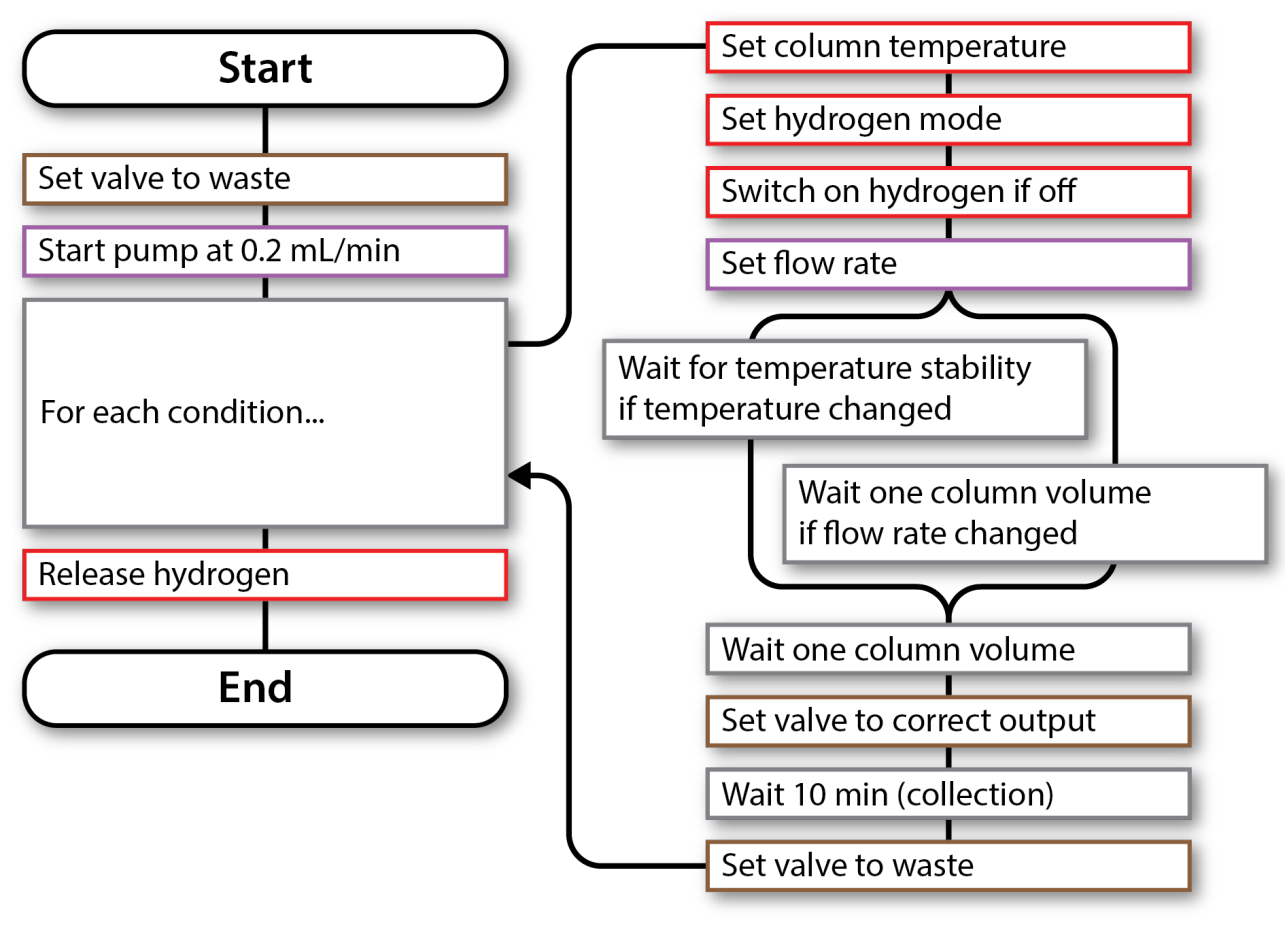
Representation of the control sequence for running experiments under a set of conditions. (See Supporting Information File 2 for control program).

Combining the control of a Knauer HPLC pump, the H-Cube^®^ reactor and a multi-position valve (**V2**) (Figure 8, Figure 9) we could perform up to nine reactions in a row. A sample was taken at steady state for each set of conditions: we noticed two major reduction products, fully-reduced pyrazine-2-carboxamide (**1**) and 1,4,5,6-tetrahydropyrazine-2-carboxamide (**4**). Unfortunately, the carbonyl stretching frequencies of the different amides proved to be remarkably similar and the secondary frequencies had very low intensities, so the FlowIR™ was not suitable for analysis of this reaction mixture and these were subsequently analysed by NMR to quantify the results.

**Figure 10:**
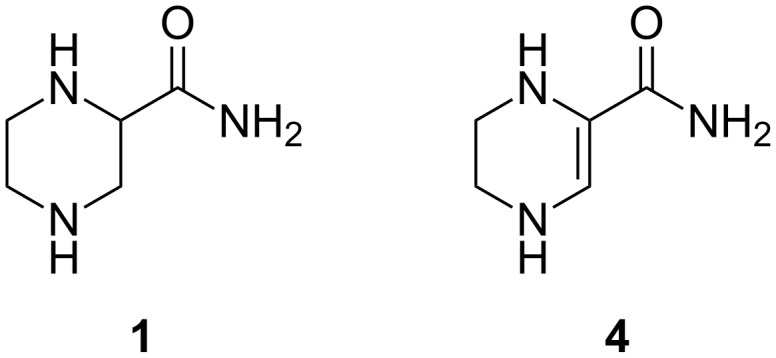
Reduction products of piperazine-2-carboxamide.

For each sample, four parameters were calculated: the degree of conversion, based on the residual **2** observed; the amount of the desired product **1** formed; and an estimated amount of undesired compounds, based on the integration of peaks visible in the ^1^H NMR spectra (a triplet at 3.2 ppm and a doublet at 3.8 ppm, the first corresponding to **4** and the other to a second unidentified compound) relative to the integration of **1** and **2** (Figure 10). As expected, the hydrogen pressure had the most significant effect on the conversion. The temperature and the flow rate had a lower effect on the conversion, although the combination of higher temperatures and lower flow rates gave a higher purity output as intermediates such as **4** are fully reduced. For maximum efficiency further hydrogenation procedures were carried out at the lower flow rate 0.1 mL min^−1^ and higher temperature 100 °C.

### In situ generation of the intermediate

Performing an optimisation experiment in continuous flow – such as described in the previous section – has a notable disadvantage. After making a change to the reaction conditions there is some delay before the system settles to a steady state, at which point a new measurement can be taken. This is particularly relevant for reactions that take some time, as large quantities of material may be required to perform a number of different trials. In the previous case, we had a significant amount of the intermediate so this was not considered to be a problem, but we would like to avoid stockpiling of intermediates in this way. One major advantage of continuous processing is that material can be used directly from one step to another so that collecting large quantities of intermediates can be avoided.

Consequently, we started to investigate whether the two steps could be combined so that there is always enough feedstock to run the second step. Normally, this would dictate that the flow rate of the second step is always the same as that of the first step (or greater, using an additional pump). However, in some processes, such as the one described in the previous section, we would like to be able to vary the flow rate of a step to adjust the residence time. We envisaged the use of a reservoir to keep a small amount of intermediate ready for the second step. This required some means to measure the volume present in the reservoir so that the control software can decide whether there is enough material ready to perform an experiment.

**Figure 11:**
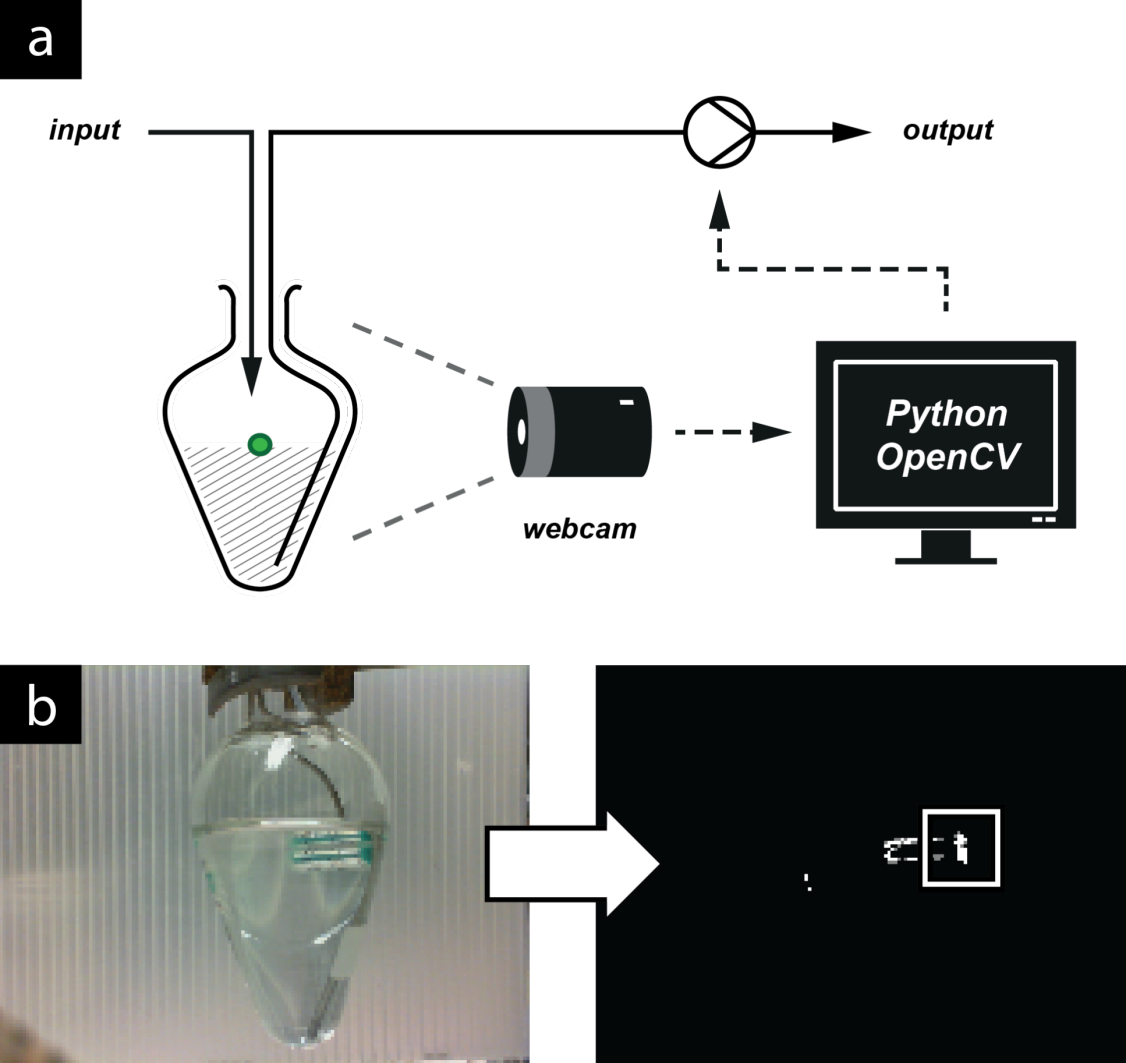
(a) In-line reservoir schematic. The liquid level is measured by observation of a plastic float. (b) Image processing to measure the liquid level. The computer receives an image of the reservoir and processes it as follows: (1) Separate the image into red, green and blue components (“channels”) and then identify the green pixels by subtracting the red channel from the green. (2) Convert the resulting greyscale image to black and white based on a certain threshold of lightness. (3) Find the centre of the largest white region (shown highlighted with a box) and report its height from the bottom of the image.

In a recent review [11], we described the application of cameras and computer vision to synthesis procedures. Building on work in which a camera and a float were employed to measure the position of a biphasic (aqueous/organic) mixture within a settling column [5], we manufactured a float containing an air bubble [39] which would float on less dense solvents than the solid polyethylene version used previously. Using a pear-shaped flask as a reservoir, so that the inlet needle for the second pump could access as much of the solution as possible, a camera/float combination was able to measure the amount of intermediate in the reservoir (Figure 11). A digital camera was positioned so that it was observing the collection vessel. Regular snapshots were taken and analysed using computer vision software to locate the green float within the image. This information was used to estimate the height of the float in the reservoir, which enabled the control protocol to make decisions based on the amount of intermediate available. The Raspberry Pi^®^ computer was found to struggle when working with a USB webcam so the control protocol was instead run on a standard desktop computer (A dedicated camera module for the Raspberry Pi^®^ has recently been released which should solve this problem as this module requires minimal processor time to capture images).

By combining the control sequences for the two synthesis steps with the volume-measuring logic, the second step could be started when enough material of the intermediate had been collected to start pumping out. Furthermore, if the meniscus were to rise high enough to pose a risk of the reservoir overflowing, this can trigger an alert or cause valve **V1** to cease collecting the intermediate. If the liquid level were to fall too low, then the next iteration of the hydrogenation condition testing loop could pause until there was enough material to continue (see Supporting Information File 3 for the control sequence, and Supporting Information File 4 for the sequence diagram).

### Two-step synthesis procedure

**Figure 12:**
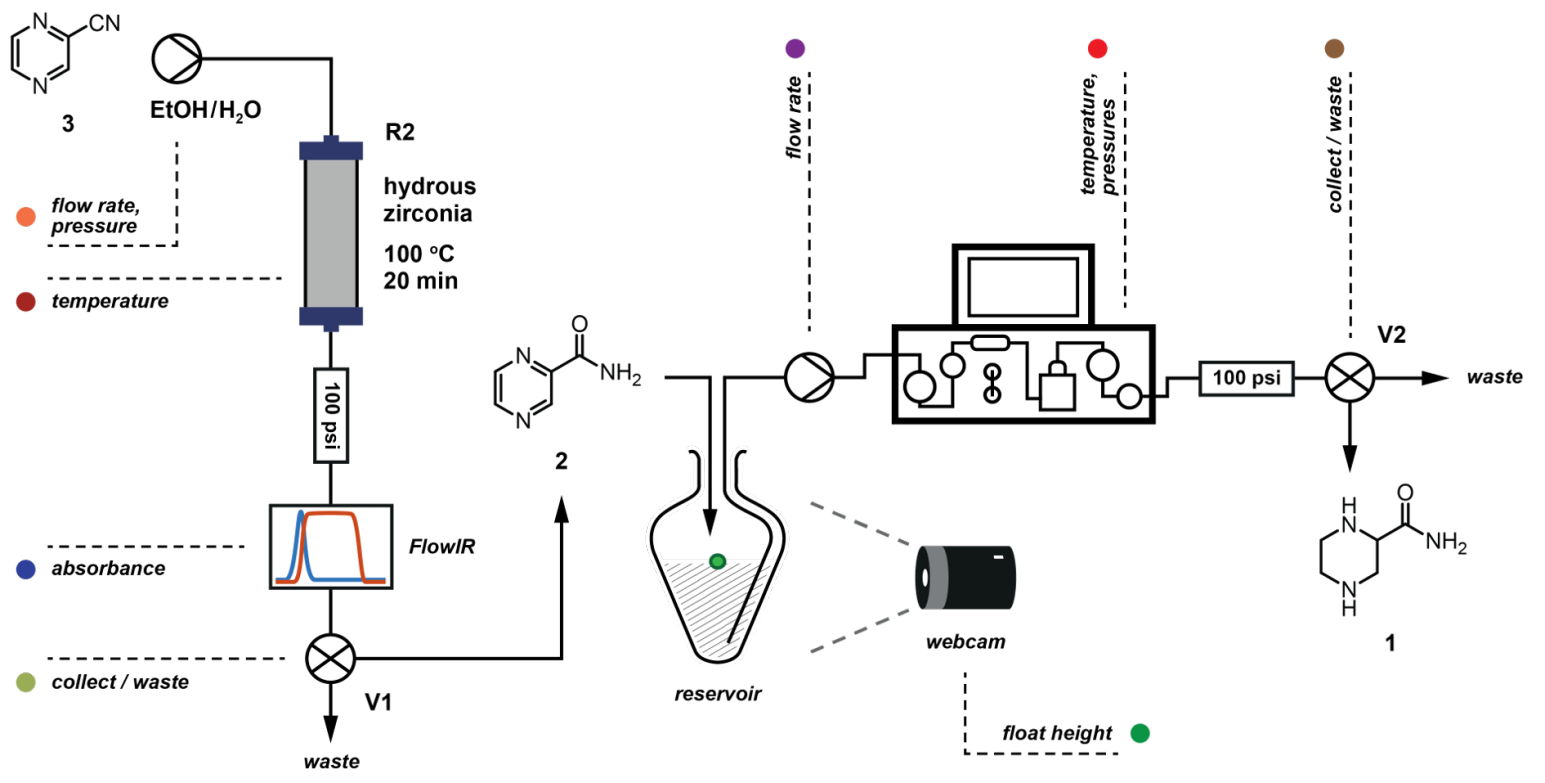
Flow set up for the automated machine assisted synthesis of (*R*,*S*)-piperidine-2-carboxamide.

**Figure 13:**
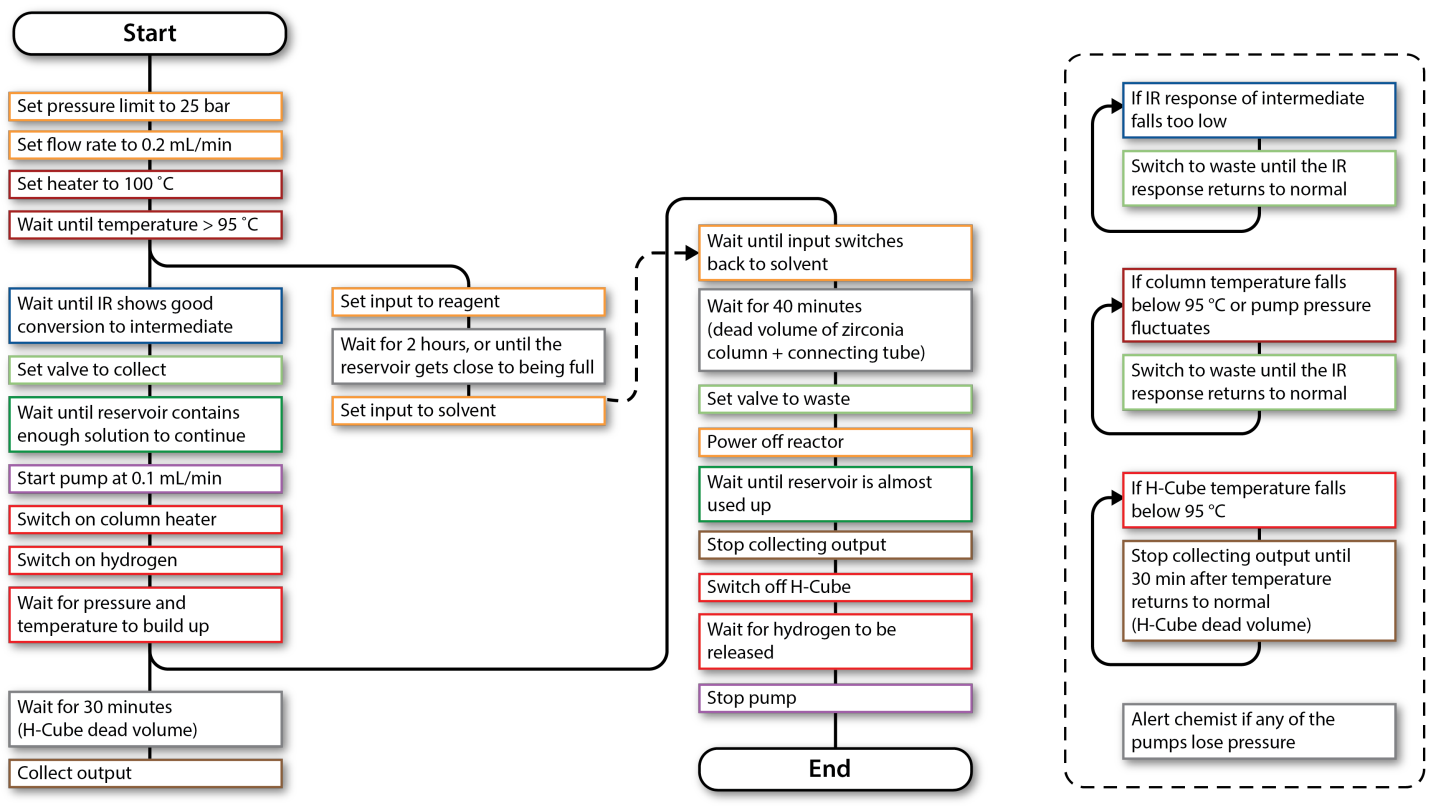
Control sequence for the two-step process.

Finally, the previous procedure was modified to perform a two-step process using the optimised parameters for each step (Figure 12, Figure 13). The maximum flow rate to allow full conversion in the H-Cube^®^ was relatively low, which limited the throughput of the material in the reduction step. This could be mitigated if larger-scale hydrogenation apparatuses were available; but in this case the use of a reservoir meant that the flow rates did not necessarily have to be matched, resulting in a semi-continuous process.

**Figure 14:**
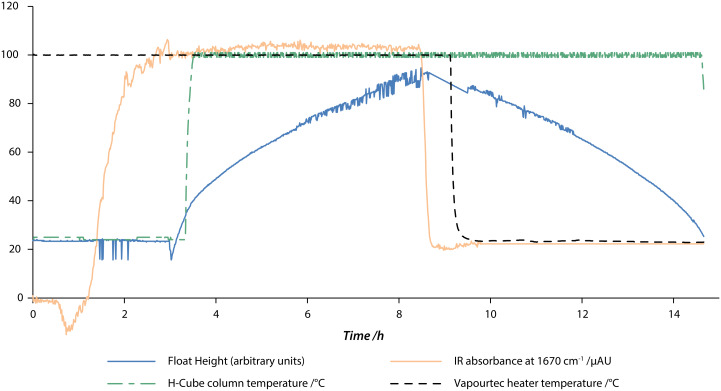
Chart of monitored parameters over a 15 hour reaction. The output from the hydrolysis step is directed into the reservoir as soon as the IR absorption crosses a threshold. When enough material has been collected, the H-Cube^®^ is powered on and the hydrogenation step is started. When the reservoir fills up the first step is stopped. The hydrogenation continues until the intermediate has been used up.

Using the two different flow rates and having the hydrolysis step stop automatically when the reservoir filled up led to a total running time of about 10 hours (Figure 14). However, we can imagine that this could represent one cycle of a prolonged sequence where the first step is periodically stopped to allow time for the collected intermediate to be processed in the second step. With a large reservoir (for example, our 50 mL reservoir allows up to a 16 hour start/stop cycle) the proportion of material wasted during start up and shutdown of the first step can be reduced.

We anticipate that the potential to rapidly realise complex control sequences such as these will further broaden the scope for the use of flow chemistry reactors in both the research and scale-up environments. We hope that the increasing availability of free and open software to enable such processes will help to democratise the field of flow chemistry when applied as an advanced enabling technology in the laboratory.

## Conclusion

A machine assisted synthesis of pyrazine-2-carboxamide – a component of Rifater^®^, used in the treatment of tuberculosis – and its reduced derivative (*R,S*)-piperazine-2-carboxamide has been demonstrated, using a new open-source software platform for the simultaneous control of multiple devices. The protocol developed here represents a valid example of how these technologies can be used to implement chemistry processes for synthesis. Automated procedures can have a significant impact on productivity, not just for traditional applications such as library synthesis, but also for one-off protocols for which automation may previously have required a much greater time investment.

## Supporting Information

File 1 Experimental data.

File 2 Control sequence for extended period hydrolysis experiment with monitoring. Alternate web version: http://gist.github.com/richardingham/0a58a291bad2e3b9009f

File 3 Control sequence for performing DoE experiments. Alternate web version: http://gist.github.com/richardingham/83401127622036c6afd0

File 4 Control sequence for performing DoE experiments using intermediate from a reservoir. Alternate web version: http://gist.github.com/richardingham/f2117b9dc7504d6e1942

File 5 Flow chart representation of the control sequence for performing DoE experiments using intermediate from a reservoir.

File 6 Control sequence for performing two-step hydrogenation process with control and monitoring. Alternate web version: http://gist.github.com/richardingham/31f6f8efa47771c2ed02
