# Supplementary material for: Integration of enabling methods for the automated flow preparation of piperazine-2-carboxamide
Source: Beilstein J Org Chem. 2014 Mar 12;10:641–52. doi: 10.3762/bjoc.10.56 (PMC3999859; doi:10.3762/bjoc.10.56)
Supplement: File 1 — Experimental data. [file Beilstein_J_Org_Chem-10-641-s001.pdf]

Supporting Information

for

Integration of enabling methods for the automated flow preparation of piperazine-2-carboxamide

Richard J. Ingham [1], Claudio Battilocchio [1], Joel M. Hawkins [2] and Steven V. Ley[1]*

Address: [1]Innovative Technology Centre, Department of Chemistry, University of Cambridge, Lensfield Road, CB2 1EW, Cambridge UK and [2]Pfizer Worldwide Research and Development, Eastern Point Road, Groton, CT 06340, USA

Email: Steven V. Ley - svl1000@cam.ac.uk

*Corresponding author

Experimental data

Experimental section

General chemistry

^{1}H NMR spectra were recorded on a Bruker Avance DPX-400 spectrometer with the residual solvent peak as the internal reference ($CDCl_3$ = 7.26 ppm, d_6-DMSO = 2.50 ppm). ^{1}H resonances are reported to the nearest 0.01 ppm. ^{13}C NMR spectra were recorded on the same spectrometers with the central resonance of the solvent peak as the internal reference ($CDCl_3$ = 77.16 ppm, d_6-DMSO = 39.52 ppm). All ^{13}C resonances are reported to the nearest 0.1 ppm. DEPT 135, COSY, HMQC, and HMBC experiments were used to aid structural determination and spectral assignment. The multiplicity of ^{1}H signals are indicated as: s = singlet, d = doublet, dd = doublet of doublet, ddd = doublet of doublet of doublet, t = triplet, q = quadruplet, sext = sextet, m = multiplet, br. = broad, or combinations of thereof. Coupling constants (*J*) are quoted in Hz and reported to the nearest 0.1 Hz. Where appropriate, averages of the signals from peaks displaying multiplicity were used to calculate the value of the coupling constant. Infrared spectra were recorded neat on a PerkinElmer Spectrum One FT-IR spectrometer using Universal ATR sampling accessories. Unless stated otherwise, reagents were obtained from commercial sources and used without purification. Hydrous zirconia was kindly gifted from MEL Chemicals (cod. XZO 631/01) [1]. The removal of solvent under reduced pressure was carried out on a standard rotary evaporator. Melting points were performed on a Stanford Research Systems MPA100 (OptiMelt) automated melting point system and are uncorrected. High resolution mass spectrometry (HRMS) was performed using a Waters Micromass LCT Premier™ spectrometer using time of flight with positive ESI, or conducted by Mr Paul Skelton (Department of Chemistry, University of Cambridge) on a Bruker BioApex 47e FTICR spectrometer using (positive) ESI or EI at 70 ev to within a tolerance of 5 ppm of the theoretically calculated value. Two FlowIR™ spectrometers (silicon and diamond window respectively) from Mettler Toledo were used for the in-line analyses of the two steps [2]. BET analyses were performed using a Tristar 3000 apparatus (Micromeritics) [3] at the Department of Material Sciences and Metallurgy, University of Cambridge. The flow hydration reaction was performed using a Vapourtec R2+/R4 flow platform [4]. A Knauer K-120 HPLC pump [5] was used for the hydrogenation step, in combination with a ThalesNano H-Cube® reactor [6].

Flow procedure for the synthesis of pyrazine-2-carboxamide. A solution of nitrile **3** in ethanol/H_2O (0.6 M, 8:1 v/v) was passed through the column reactor **R2** (100 mm × 10 mm, 5 g hydrous zirconia) heated at 100 °C, with a residence time of 20 minutes, to obtain a quantitative recovery of the primary amide **2** after concentration of the reactor output (>98% yield). White solid; m.p. 191–194 °C; δ H (400 MHz, d_6-DMSO, 25 °C) 7.84 (1H, br. s), 8.24 (1H, br. s), 8.70 (1H, dd, *J* = 2.5 Hz, J 1.5 Hz), 8.85 (1H, d, *J* = 2.5 Hz), 9.17 (1H, d, *J* = 1.5 Hz); δ C (100 MHz, $CDCl_3$, 25 °C) 143.46 (CH), 143.69 (CH), 145.18 (C), 147.46 (CH), 165.13 (C); FTIR (neat, *v*): 3422, 3132, 1669, 1583, 1525, 1481, 1432, 1373, 1171, 1089, 1046, 1021, 870, 791 cm^{-1}; LC-MS: retention time 0.28 min, *m/z* $[M + H]^+$ = 124.19; HRMS (ESI): *m/z* calcd for $C_5H_6ON_3^+$: 124.0505; found 124.0504. Elemental analysis: calcd C = 48.78%, H = 4.09%, N = 34.13%; found C = 48.60%, H = 4.19%, N = 33.70%.

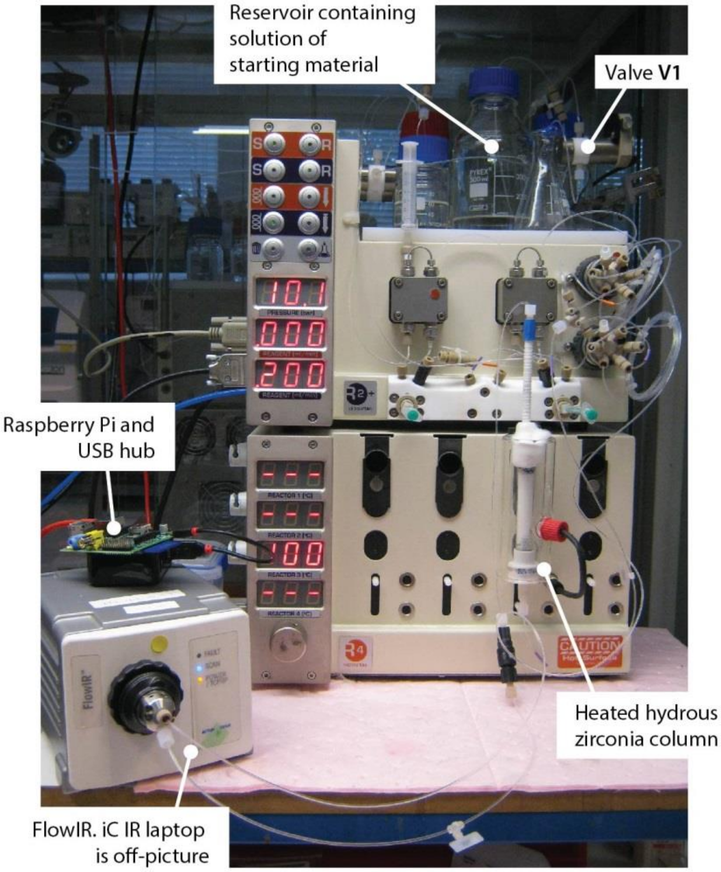

Using a single stream of a Vapourtec R2/R4+ reactor, material is pumped through a polymer tubing to the glass column reactor. The output of this reactor passes through a second tubing to a 100 psi back-pressure regulator (BPR) and then through a third tubing to the switching valve **V1**, directing it either to waste or to be collected. All tubing is PFA with Ø 1mm.

Flow procedure for the synthesis of (*R,S*)-piperazine-2-carboxamide. A solution of carboxamide **2** in ethanol/H_2O (0.6 M, 8:1 v/v) was fed using a Knauer K-120 pump (flow rate 0.1 mL min^{-1}) into the H-Cube apparatus, loaded with a 10% Pd/C catalyst cartridge, heated at 100 °C to obtain a quantitative

transformation to the primary amide **1** after concentration of the reactor output (95% yield). White solid; δ H (400 MHz, MeOD, 25 °C) ^{1}H NMR (400 MHz, MeOD) δ 2.66 (2H, ddd, *J* = 16.3, 7.7, 4.3 Hz), 2.80 – 2.71 (1H, m), 2.82 (1H, dt, *J* = 11.8, 2.7 Hz), 2.95 (1H, dt, *J* = 12.2, 2.7 Hz), 3.08 (1H, dd, *J* = 12.4, 3.4 Hz), 3.36 – 3.28 (2H, m); δ C (100 MHz, $CDCl_3$, 25 °C) 45.56 (CH_2), 46.26 (CH_2), 49.63 (CH_2), 59.39 (CH), 174.58 (C); FTIR (neat, *v*): 3332, 3308, 3194, 2949, 2904, 2832, 1673, 1611, 1488, 1438, 1409, 1355, 1306, 1186, 1136, 1116, 1071, 1057, 1003, 959, 908, 823, 723 cm^{-1}; LC-MS: retention time 0.26 min, *m/z* $[M + H]^+$ = 130.14; HRMS (ESI): *m/z* calcd for $C_5H_{12}ON_3^+$: 130.0975; found 130.0979. Elemental analysis: calcd C = 46.50%, H = 8.58%, N = 32.53%; found C = 46.49%, H = 8.50%, N = 32.30%.

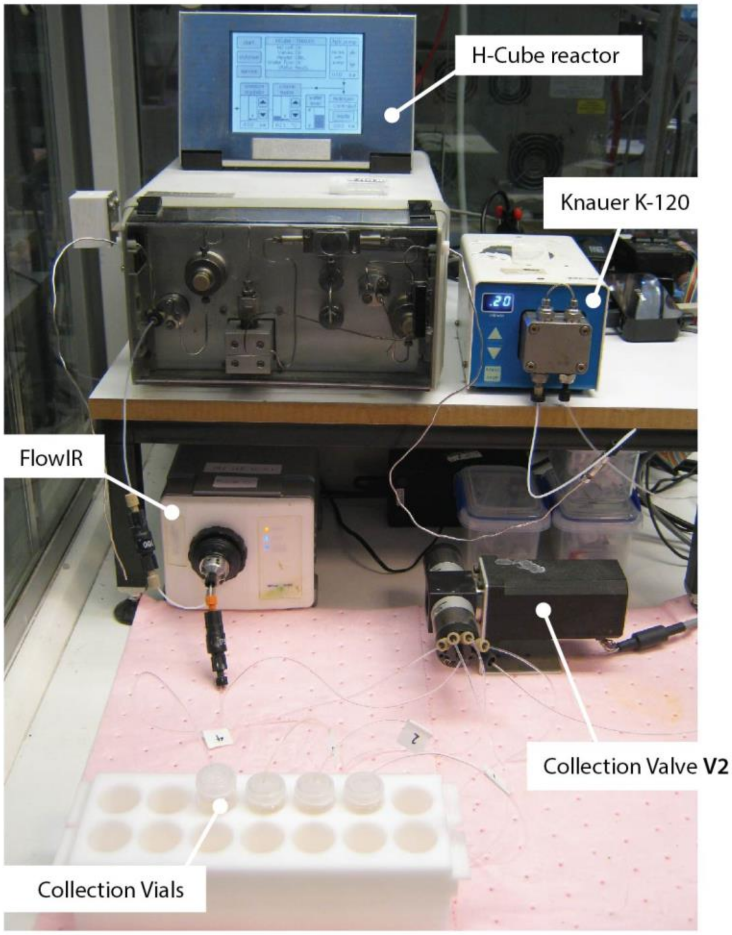

The reagent solution is infused using the Knauer K-120 pump into the H-Cube® via a Ø 0.5 mm PTFE tubing and Ø 0.5 mm stainless steel tubing. The output of the H-Cube® passes through Ø 0.5 mm PTFE tubing through a 100 psi BPR, then the FlowIR™ spectrometer, and then a 75 psi BPR to the collection valve **V2** (Valco VICI 10-position switching valve). The second BPR was required to stop the hydrogen from blowing the solution through the FlowIR™ too rapidly, whilst not providing more pressure than the IR head can withstand.

Flow procedure for the telescoped synthesis of (*R,S*)-piperazine-2-carboxamide. A solution of nitrile **3** in ethanol/H_2O (0.6 M, 8:1 v/v) was passed through the column reactor **R2** (100 mm × 10 mm, 5 g hydrous zirconia) heated at 100 °C, with a residence time of 20 minutes. This intermediate solution was

used directly without purification in the second step. This could be performed either by matching the flow rates of the two steps, or using a reservoir arrangement as described in the main article. The intermediate solution was delivered to the H-Cube® apparatus (flow rate 0.1 mL min^{-1}) using a Knauer K-120 pump. The H-Cube® was loaded with a 10% Pd/C catalyst cartridge, which was heated at 100 °C. After concentration of the reactor output the primary amide **1** was obtained (95% yield).

Collection reservoir

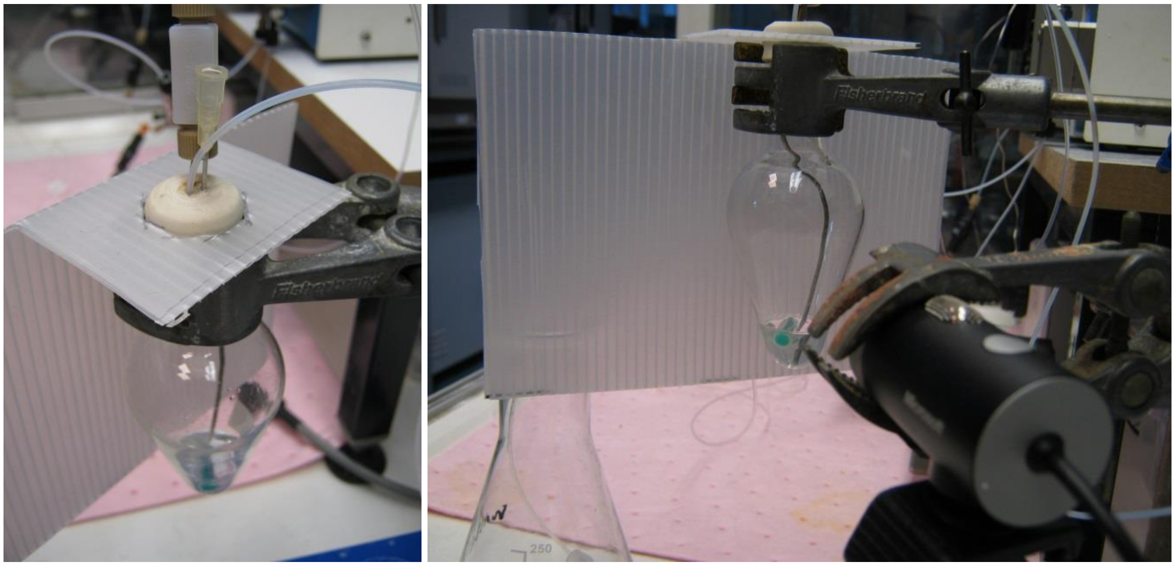

The intermediate solution was directed into a pear shaped flask through a tube (Ø 0.5 mm PTFE, total volume 1 mL) from **V1**. A bent stainless steel tube allows the solution to be drawn out by the Knauer K-120 pump. An open needle allows the pressure to equalise. A plastic board gives a white background to the image captured by the camera, which is held in position relative to the flask with clamps.

DoE run results

Run	H_2 Pressure	Temperature /°C	Flow rate /mL min^{-1}	Conversion	Product	Side-product 1	Side-product 2
**1**	Full	100	0.1	1.00	1.00	0.00	0.00
**2**	20 bar	100	0.1	0.53	0.35	0.08	0.10
**3**	20 bar	40	0.2	0.85	0.08	0.07	0.70
**4**	Full	40	0.1	0.78	0.74	0.04	0.00
**5**	20 bar	40	0.2	0.52	0.34	0.18	0.00
**6**	20 bar	40	0.1	0.38	0.26	0.12	0.00
**7**	20 bar	100	0.2	0.37	0.34	0.03	0.00
**8**	Full	40	0.2	0.80	0.54	0.26	0.00
**9**	20 bar	40	0.1	0.46	0.42	0.04	0.00
**10**	Full	100	0.1	1.00	1.00	0.00	0.00
**11**	Full	40	0.1	0.73	0.59	0.14	0.00
**12**	Full	100	0.2	1.00	1.00	0.00	0.00
**13**	Full	100	0.2	1.00	1.00	0.00	0.00
**14**	20 bar	100	0.1	0.40	0.37	0.04	0.00
**15**	Full	40	0.2	0.75	0.52	0.23	0.00
**16**	20 bar	100	0.2	0.44	0.44	0.00	0.00

Values calculated from NMR, based on relative integration of peaks at 9.24 ppm (starting material), 3.13 ppm (product), 3.79 ppm (side-product 1) and 3.18 ppm (side-product 2).

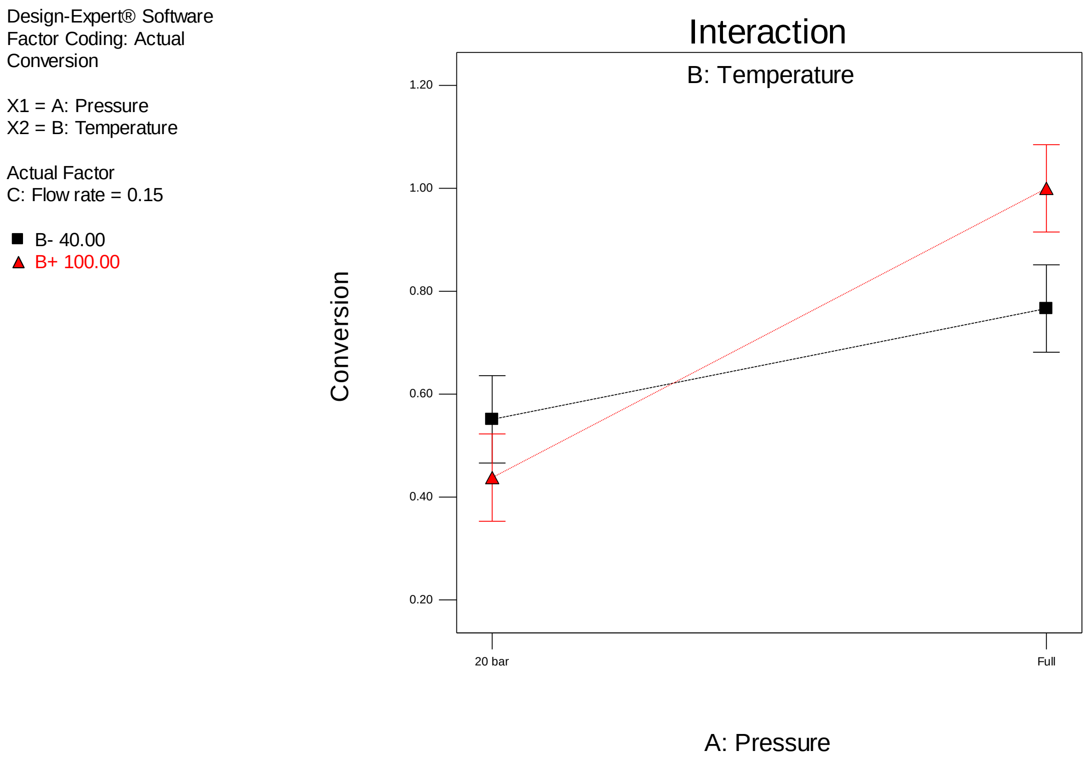

Design-Expert® Software
Factor Coding: Actual
Conversion
X1 = A: Pressure
X2 = B: Temperature
Actual Factor
C: Flow rate = 0.15
B- 40.00
B+ 100.00
Interaction
B: Temperature
Conversion
1.20
1.00
0.80
0.60
0.40
0.20
20 bar
Full
A: Pressure

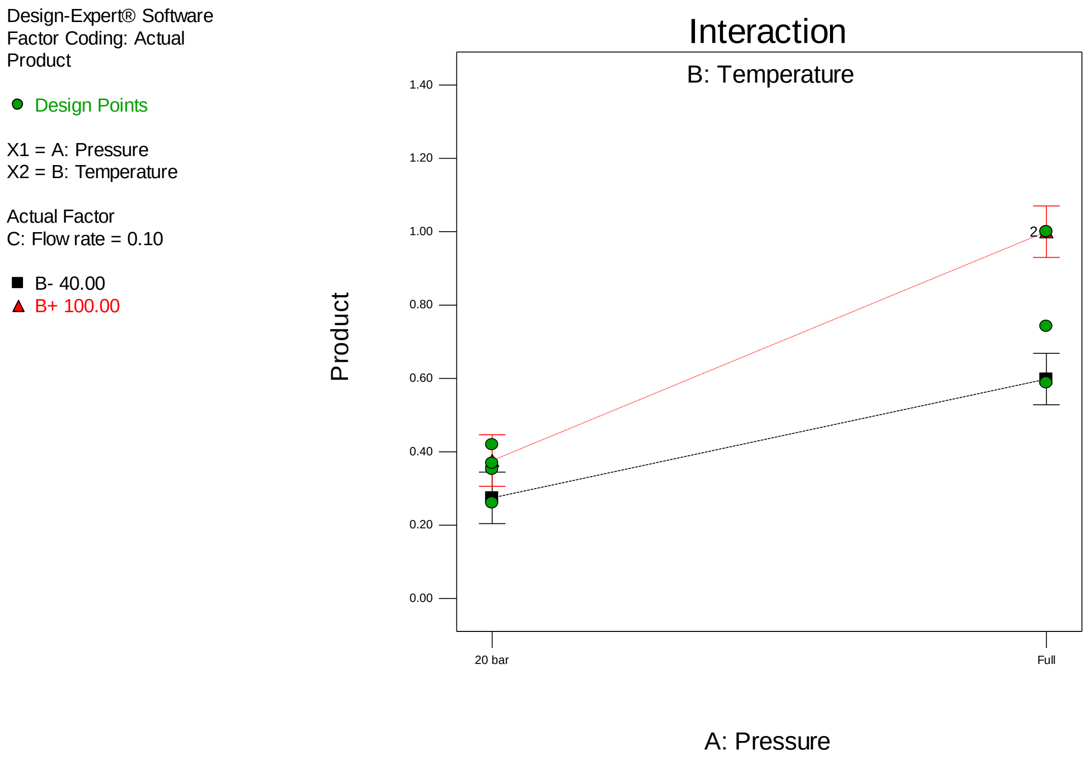

Design-Expert® Software
Factor Coding: Actual
Product
Design Points
X1 = A: Pressure
X2 = B: Temperature
Actual Factor
C: Flow rate = 0.10
B- 40.00
B+ 100.00
Interaction
B: Temperature
Product
1.40
1.20
1.00
0.80
0.60
0.40
0.20
0.00
2
20 bar
Full
A: Pressure

Design-Expert® Software
Factor Coding: Actual
SP1

● Design Points

X1 = A: Pressure
X2 = B: Temperature

Actual Factor
C: Flow rate = 0.10

■ B- 40.00
▲ B+ 100.00

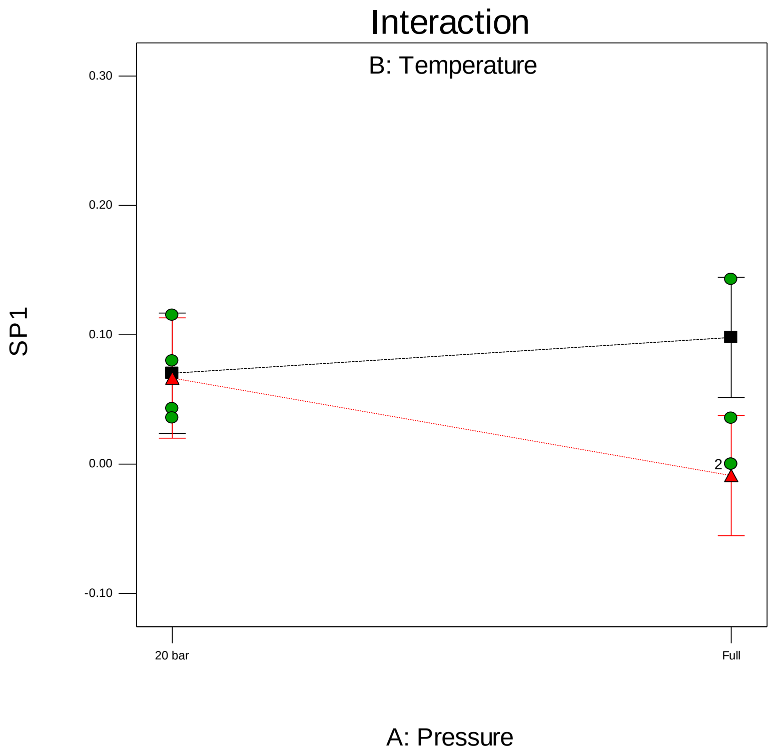

Digital Connections

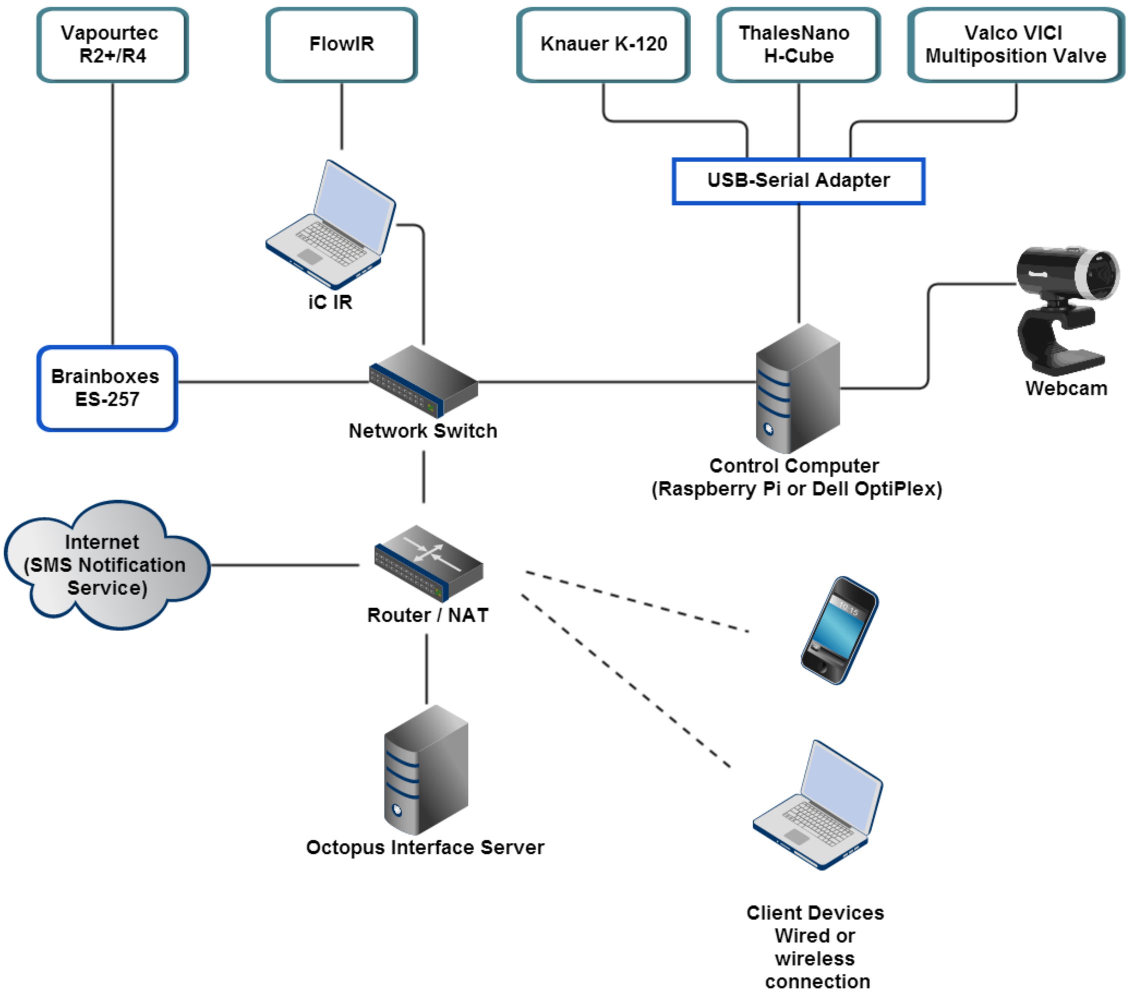

Individual devices were connected as most appropriate to the control computer. The Vapourtec unit was situated a few metres from the control computer and so an Ethernet connection was most convenient. Other devices were closer and were connected by USB or USB/Serial Adapter.

The FlowIR™ has to be controlled by the Mettler-Toledo iC IR software. This is set to perform an auto-export of data to a text file. A small script running on the laptop makes this data accessible to the control computer.

The interface server can be the same machine as the control computer. In this case it was a separate machine outside the lab. The server software can also be run on a virtual machine in the cloud allowing internet access to the experimental data. Importantly the control computer can be behind a firewall and not visible from the internet, increasing the security of the laboratory devices.

This same configuration was used for all of the experiments; a Raspberry Pi® [7] computer was used for experiments not involving a camera.

BET measurements data for the hydrous zirconia catalyst (cod. XZO631/01, MEL Chemicals).

Isotherm Tabular Report

Relative Pressure (P/Po)	Absolute Pressure (mmHg)	Quantity Adsorbed (cm^3/g STP)	Elapsed Time (h:min)	Saturation Pressure (mmHg)
			00:39	781.13434
0.010564699	8.25021	57.8743	01:09	
0.030148522	23.54193	68.0283	01:17	
0.060813265	47.48395	77.2127	01:24	
0.085770453	66.96606	83.1222	01:32	
0.098558777	76.94717	85.8828	01:37	
0.117520648	91.74615	89.8118	01:43	
0.137303355	107.18528	93.6985	01:48	
0.157206557	122.71706	97.4922	01:53	
0.177275022	138.37518	101.1974	01:59	
0.197381660	154.06279	104.8489	02:04	
0.248717216	194.11777	113.5836	02:12	
0.297637232	232.28601	121.1762	02:18	
0.352207416	274.85944	128.2265	02:24	
0.397142912	309.91544	132.6440	02:28	
0.447467828	349.17444	136.0868	02:32	
0.497209032	387.97864	138.5256	02:35	
0.547574017	427.27133	140.4535	02:37	
0.597697991	466.37454	142.1346	02:39	
			02:40	780.27753
0.648074148	505.67145	143.7969	02:42	
0.697307297	544.07977	145.5344	02:44	
0.746892425	582.76178	147.5260	02:46	
0.796559342	621.50281	150.0012	02:49	
0.818991347	638.99713	151.3472	02:51	
0.847971249	661.59570	153.3857	02:54	
0.872693282	680.87567	155.5836	02:56	
0.896994950	699.82288	158.4901	02:59	
0.921022295	718.55536	162.5055	03:02	
0.950040325	741.16235	171.1658	03:09	
0.972998912	759.03571	186.6551	03:17	
0.980025736	764.49371	197.0335	03:22	
0.991029177	773.07245	212.1142	03:23	
0.991359953	773.32092	218.5023	03:25	
0.989001368	771.47632	208.7006	03:26	
0.976407608	761.63837	202.5224	03:29	
0.970459723	756.98474	197.0186	03:32	
0.951426327	742.10150	182.1891	03:40	
0.927109244	723.11212	171.0871	03:45	
0.892979680	696.46216	162.1981	03:52	
0.867330651	676.44098	158.2733	03:56	
0.839817773	654.97528	155.2783	03:58	
0.827915726	645.68488	154.1757	04:00	
0.802199907	625.62158	152.3462	04:02	
0.754293518	588.24933	149.6367	04:05	
0.703653787	548.75024	147.3666	04:07	
0.653385425	509.54175	145.4824	04:09	
0.603061856	470.29117	143.7837	04:11	
0.552562203	430.90158	142.2303	04:14	
0.502505861	391.86157	140.7400	04:16	
0.453860645	353.92075	138.6243	04:19	
0.405920740	316.52740	134.7340	04:24	

Isotherm Tabular Report

Relative Pressure (P/Po)	Absolute Pressure (mmHg)	Quantity Adsorbed (cm³/g STP)	Elapsed Time (h:min)	Saturation Pressure (mmHg)
0.354818260	276.67035	129.2679	04:29	
0.304357259	237.31447	122.8228	04:35	
			04:40	779.69928
0.255522172	199.23045	115.2801	04:41	
0.204987040	159.82825	106.4465	04:49	
0.145712509	113.61194	95.3960	04:56	

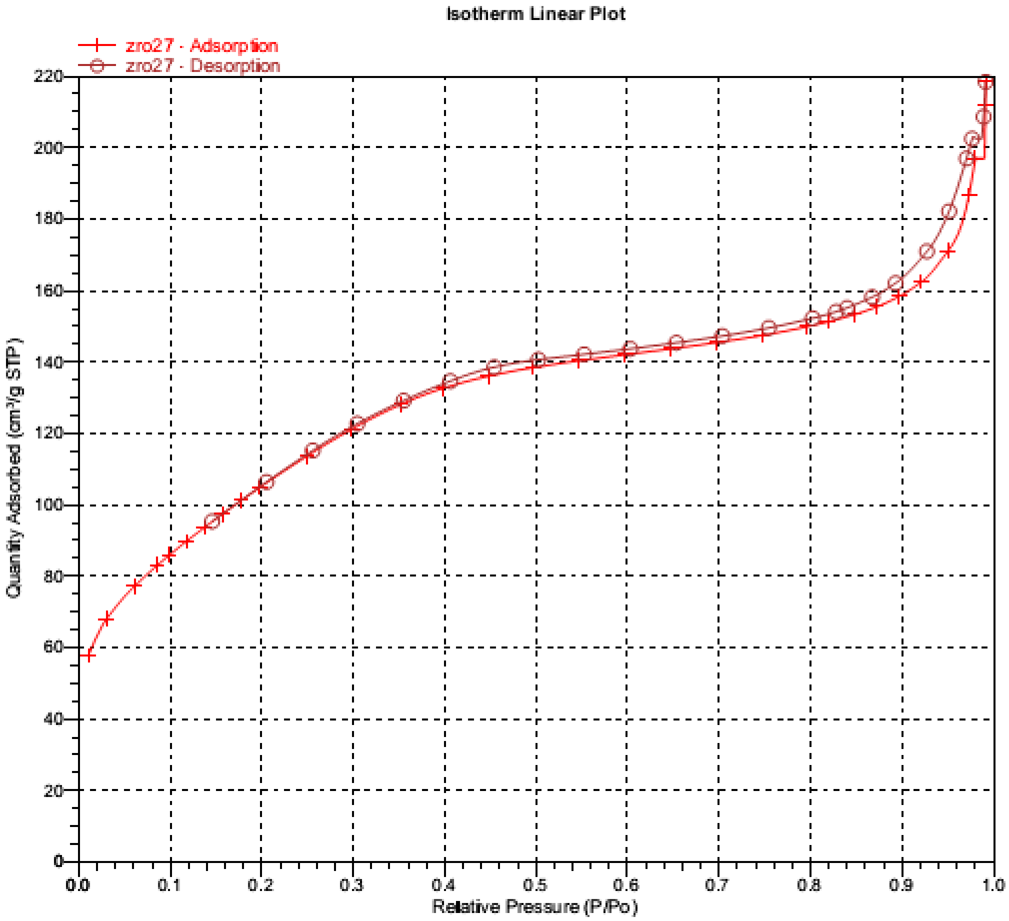

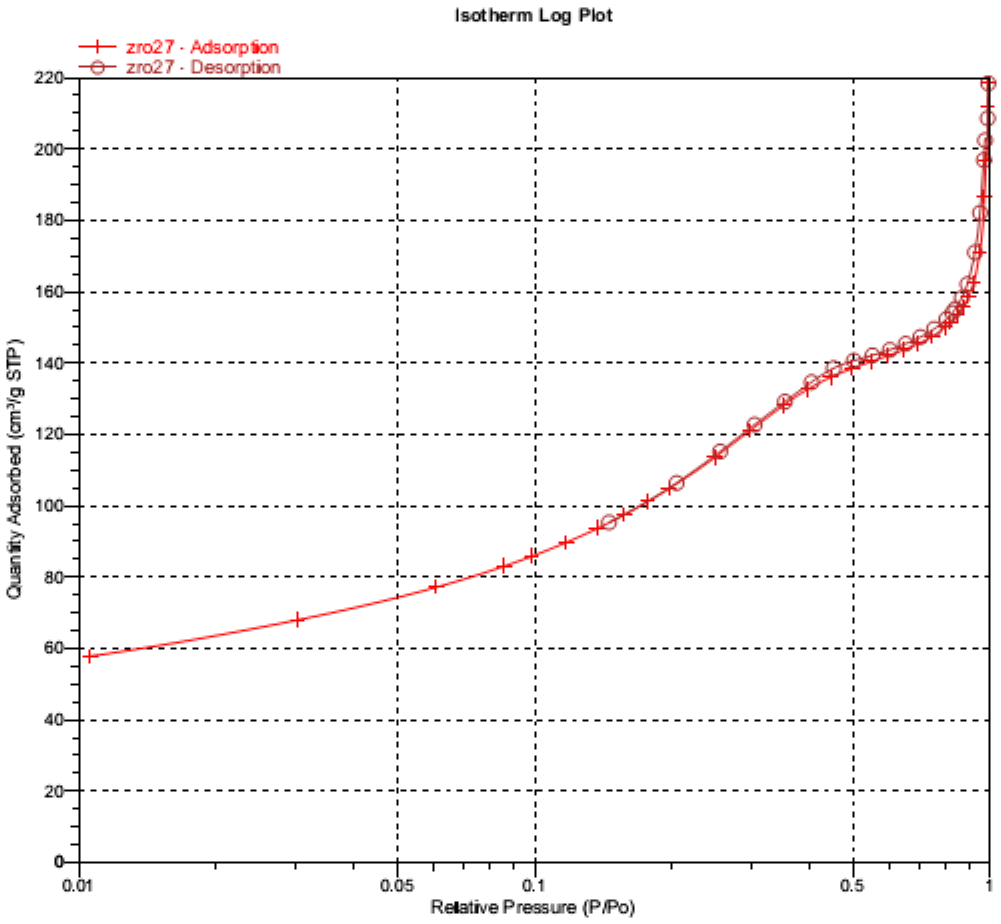

BET Surface Area Report

BET Surface Area: 376.5479 ± 5.2146 m²/g
Slope: 0.011443 ± 0.000159 g/cm³ STP
Y-Intercept: 0.000118 ± 0.000019 g/cm³ STP
C: 98.134312
Qm: 86.4991 cm³/g STP
Correlation Coefficient: 0.9992294
Molecular Cross-Sectional Area: 0.1620 nm²

Relative Pressure (P/Po)	Quantity Adsorbed (cm³/g STP)	1/[Q(Po/P - 1)]
0.010564699	57.8743	0.000184
0.030148522	68.0283	0.000457
0.060813265	77.2127	0.000839
0.085770453	83.1222	0.001129
0.098558777	85.8828	0.001273
0.117520648	89.8118	0.001483
0.137303355	93.6985	0.001699
0.157206557	97.4922	0.001913
0.177275022	101.1974	0.002129
0.197381660	104.8489	0.002345

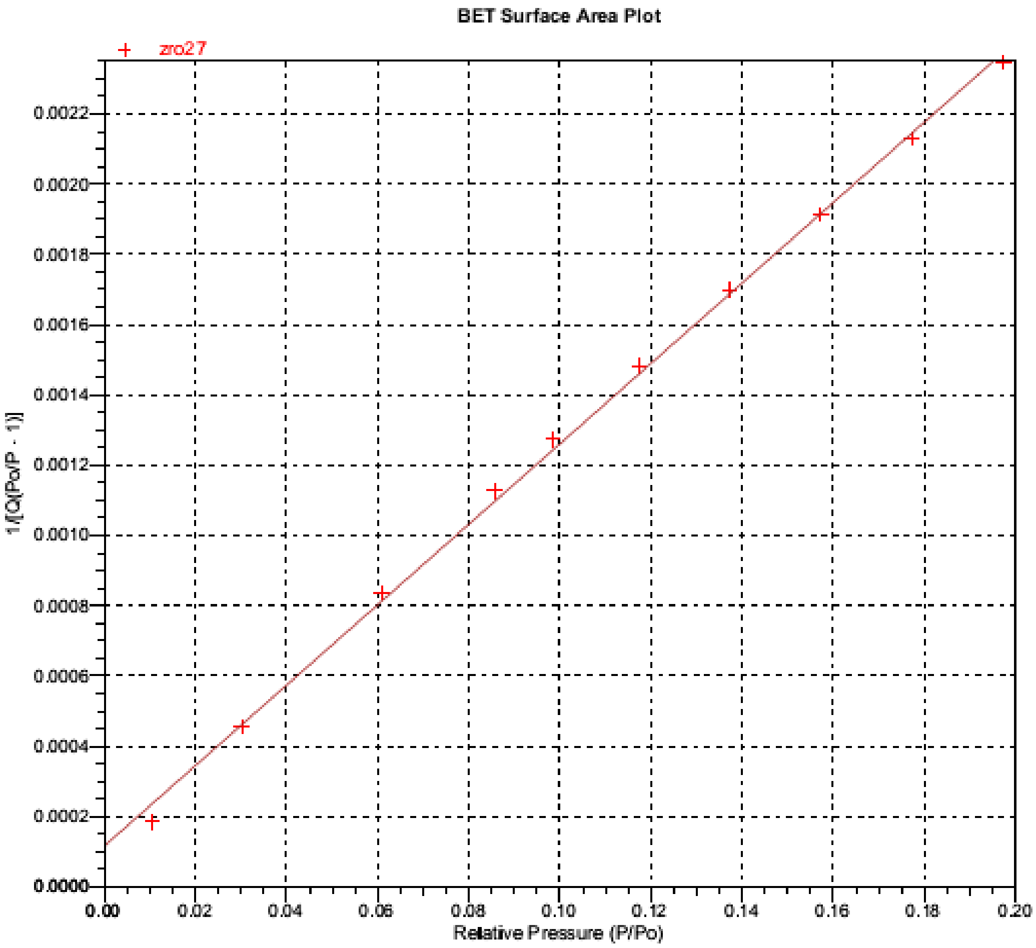

Langmuir Surface Area Report

Langmuir Surface Area: 485.0454 ± 19.6167 m²/g
Slope: 0.008975 ± 0.000363 g/cm³ STP
Y-Intercept: 0.155174 ± 0.034650 mmHg·g/cm³ STP
b: 0.057837 1/mmHg
Qm: 111.4227 cm³/g STP
Correlation Coefficient: 0.993521
Molecular Cross-Sectional Area: 0.1620 nm²

Pressure (mmHg)	Quantity Adsorbed (cm³/g STP)	P/Q (mmHg·g/cm³ STP)
8.25021	57.8743	0.143
23.54193	68.0283	0.346
47.48395	77.2127	0.615
66.96606	83.1222	0.806
76.94717	85.8828	0.896
91.74615	89.8118	1.022
107.18528	93.6985	1.144
122.71706	97.4922	1.259
138.37518	101.1974	1.367
154.06279	104.8489	1.469

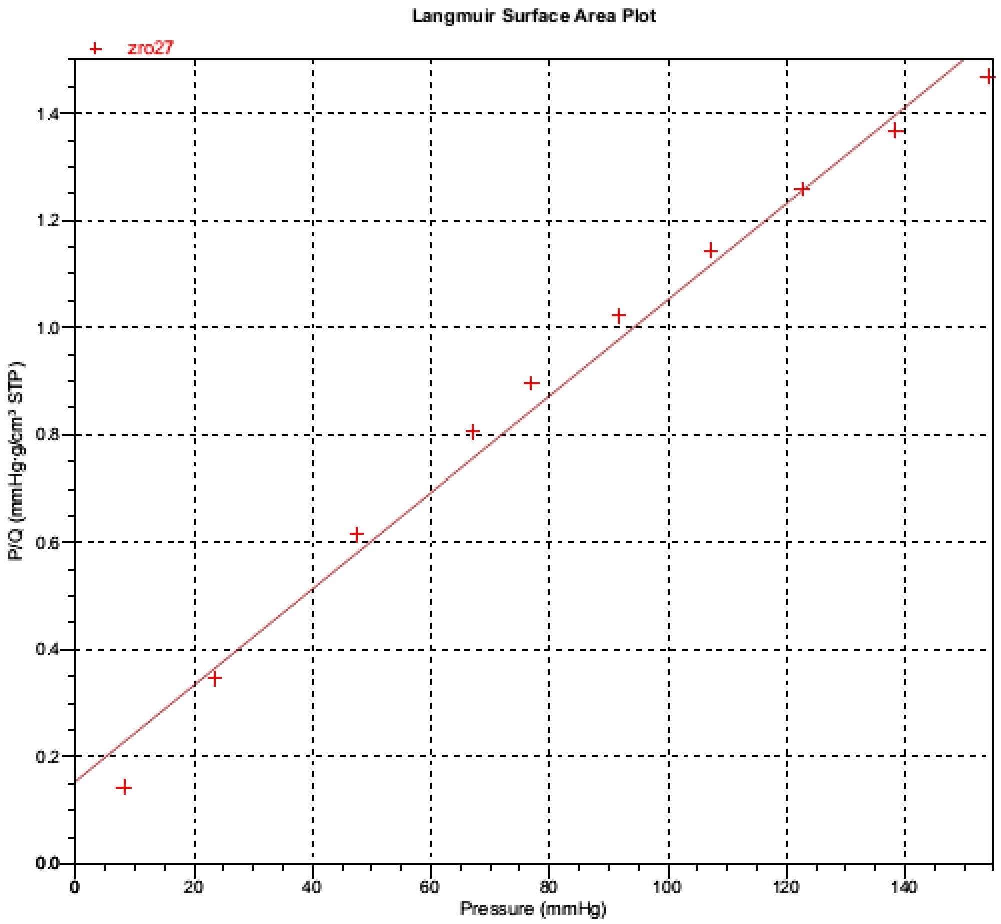

Langmuir Surface Area Plot
zro27
P/Q (mmHg·g/cm³ STP)
0.0
0.2
0.4
0.6
0.8
1.0
1.2
1.4
0
20
40
60
80
100
120
140
Pressure (mmHg)

t-Plot Report

Micropore Volume: -0.019061 cm³/g STP
Micropore Area: *
External Surface Area: 415.0610 m²/g
Slope: 268.335260 ± 2.476795 cm³/g·nm STP
Y-Intercept: -12.322642 ± 1.033230 cm³/g STP
Correlation Coefficient: 0.999702
Surface Area Correction Factor: 1.000
Density Conversion Factor: 0.0015468
Total Surface Area (BET): 376.5479 m²/g
Thickness Range: 0.35000 nm to 0.50000 nm
Thickness Equation: Harkins and Jura

t = [13.99 / (0.034 - log(P/Po))] ^ 0.5

Relative Pressure (P/Po)	Statistical Thickness (nm)	Quantity Adsorbed (cm³/g STP)
0.010564699	0.26381	57.8743
0.030148522	0.29997	68.0283
0.060813265	0.33454	77.2127
0.085770453	0.35652	83.1222
0.098558777	0.36671	85.8828
0.117520648	0.38097	89.8118
0.137303355	0.39507	93.6985
0.157206557	0.40870	97.4922
0.177275022	0.42206	101.1974
0.197381660	0.43519	104.8489
0.248717216	0.46816	113.5836
0.297637232	0.49968	121.1762
0.352207416	0.53586	128.2265
0.397142912	0.56707	132.6440
0.447467828	0.60419	136.0868
0.497209032	0.64387	138.5256
0.547574017	0.68800	140.4535
0.597697991	0.73706	142.1346
0.648074148	0.79317	143.7969

* The micropore area is not reported because either the micropore volume is negative or the calculated external surface area is larger than the total surface area.

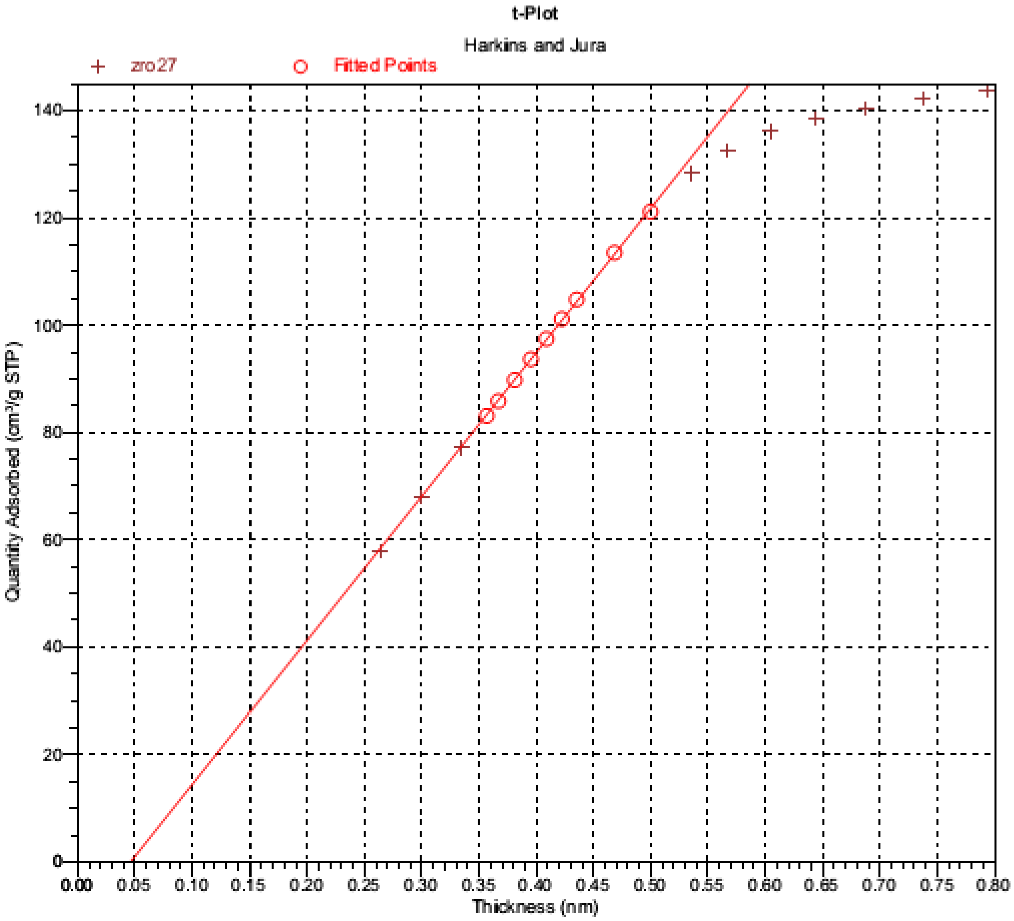

t-Plot
Harkins and Jura
zro27
Fitted Points
Quantity Adsorbed (cm³/g STP)
Thickness (nm)

BJH Adsorption Pore Distribution Report

t = 3.54 [-5 / ln(P/Po)] ^ 0.333

Diameter Range: 1.7000 nm to 300.0000 nm
Adsorbate Property Factor: 0.95300 nm
Density Conversion Factor: 0.0015468
Fraction of Pores Open at Both Ends: 0.00

Pore Diameter Range (nm)	Average Diameter (nm)	Incremental Pore Volume (cm^3/g)	Cumulative Pore Volume (cm^3/g)	Incremental Pore Area (m^2/g)	Cumulative Pore Area (m^2/g)
225.5 - 217.3	221.3	0.010421	0.010421	0.188	0.188
217.3 - 98.9	118.7	0.025039	0.035460	0.844	1.032
98.9 - 73.6	82.5	0.017498	0.052958	0.848	1.880
73.6 - 40.4	47.8	0.026795	0.079753	2.240	4.121
40.4 - 25.9	30.0	0.015139	0.094892	2.017	6.138
25.9 - 20.1	22.2	0.007005	0.101897	1.261	7.399
20.1 - 16.3	17.8	0.005125	0.107022	1.152	8.551
16.3 - 13.8	14.8	0.003894	0.110916	1.051	9.603
13.8 - 11.6	12.5	0.003659	0.114574	1.172	10.775
11.6 - 10.4	10.9	0.002458	0.117033	0.901	11.676
10.4 - 8.4	9.1	0.004661	0.121693	2.045	13.721
8.4 - 7.0	7.5	0.003852	0.125545	2.046	15.767
7.0 - 6.0	6.4	0.003501	0.129046	2.188	17.956
6.0 - 5.2	5.5	0.003532	0.132578	2.551	20.507
5.2 - 4.6	4.9	0.003848	0.136426	3.168	23.675
4.6 - 4.1	4.3	0.004885	0.141311	4.536	28.211
4.1 - 3.7	3.9	0.006928	0.148239	7.193	35.403
3.7 - 3.3	3.5	0.010912	0.159151	12.599	48.002
3.3 - 3.0	3.1	0.015348	0.174499	19.515	67.517
3.0 - 2.7	2.8	0.026238	0.200737	36.940	104.456
2.7 - 2.5	2.6	0.029646	0.230383	46.251	150.707
2.5 - 2.2	2.3	0.035009	0.265392	60.600	211.307
2.2 - 2.1	2.2	0.014672	0.280064	27.243	238.550
2.1 - 2.0	2.1	0.014781	0.294845	28.702	267.252
2.0 - 1.9	2.0	0.015025	0.309870	30.565	297.817

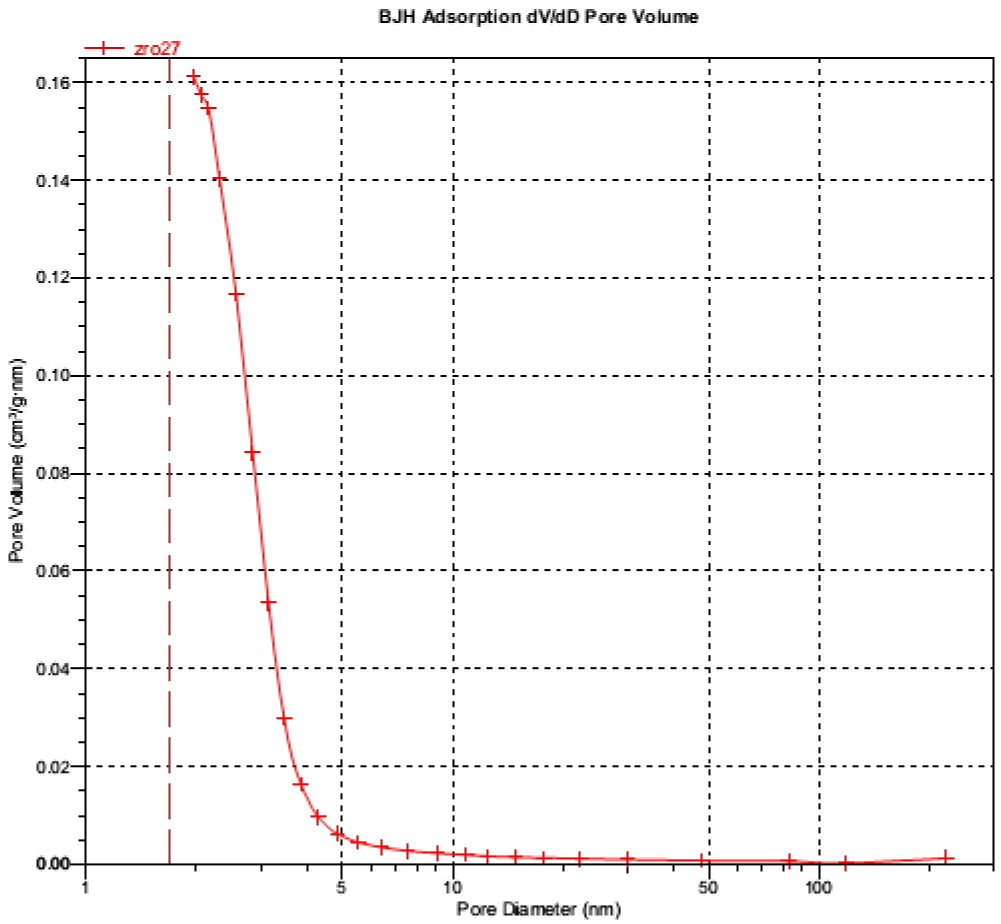

BJH Adsorption dV/dD Pore Volume
zro27
Pore Volume (cm³/g·nm)
0.16
0.14
0.12
0.10
0.08
0.06
0.04
0.02
0.00
1
5
10
50
100
Pore Diameter (nm)

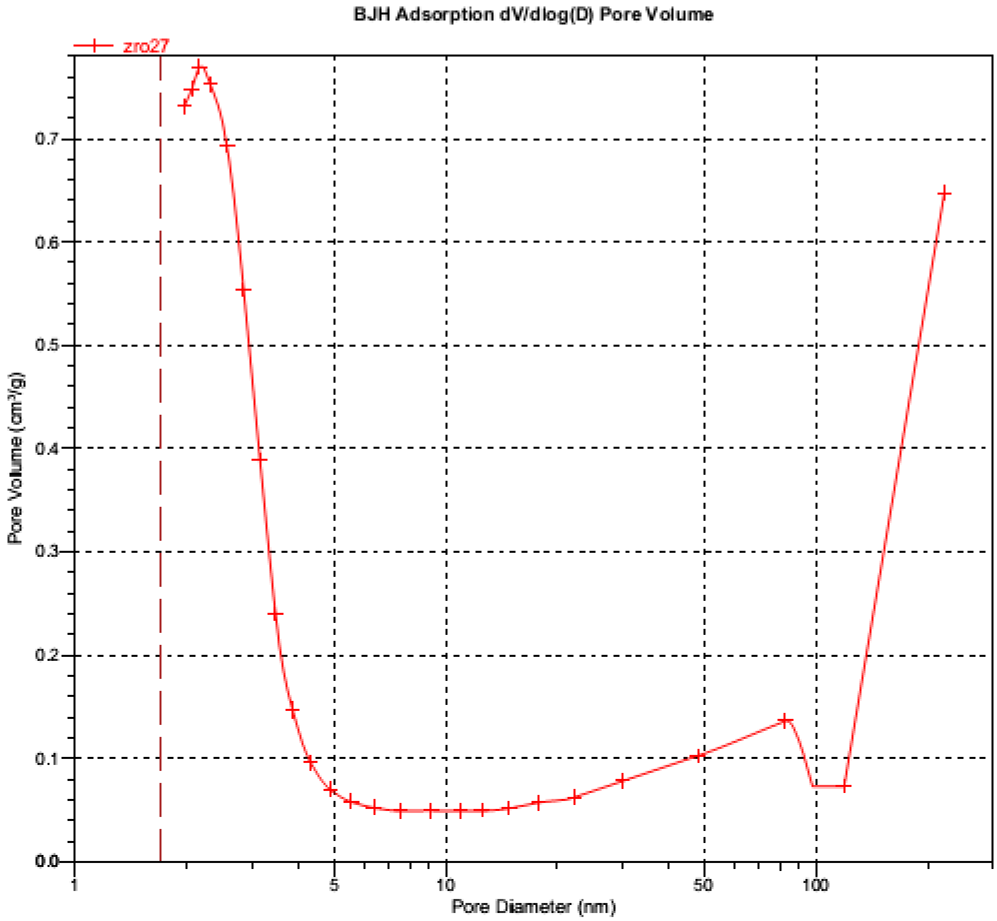

BJH Adsorption dV/dlog(D) Pore Volume
zro27
Pore Volume (cm³/g)
0.0
0.1
0.2
0.3
0.4
0.5
0.6
0.7
1
5
10
50
100
Pore Diameter (nm)

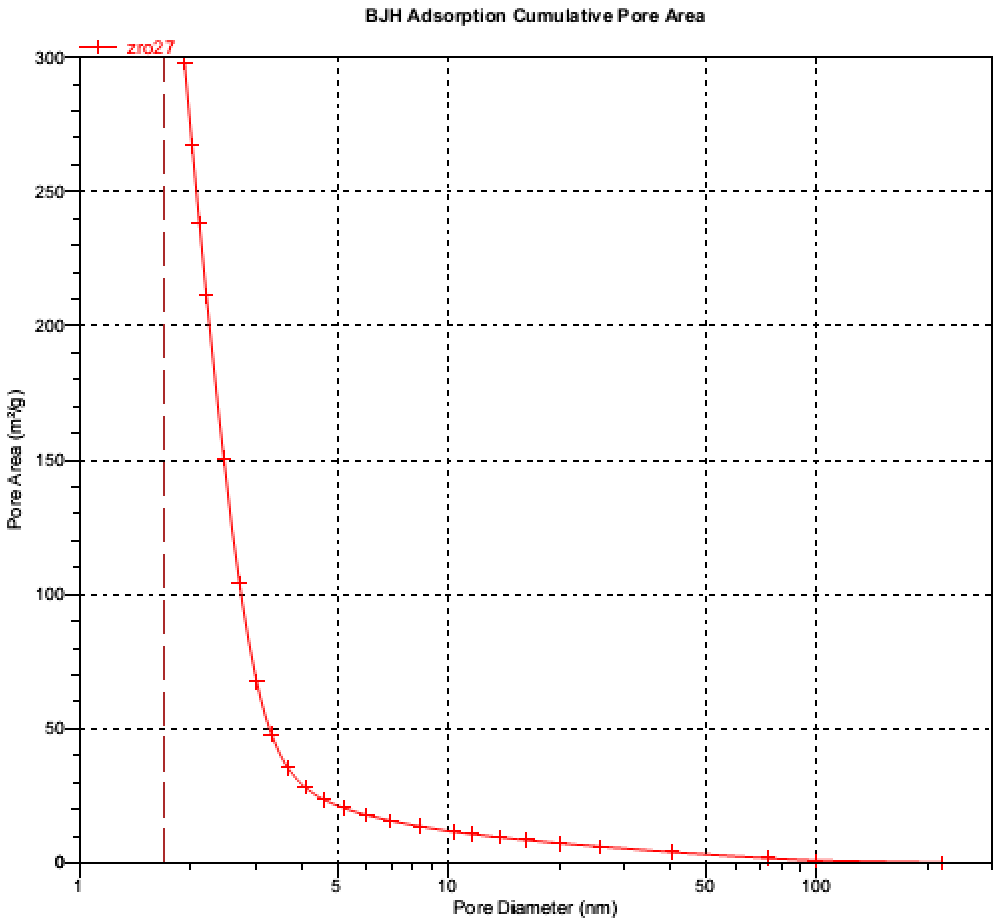
BJH Adsorption Cumulative Pore Area
zro27
Pore Area (m²/g)
300
250
200
150
100
50
0
1
5
10
50
100
Pore Diameter (nm)

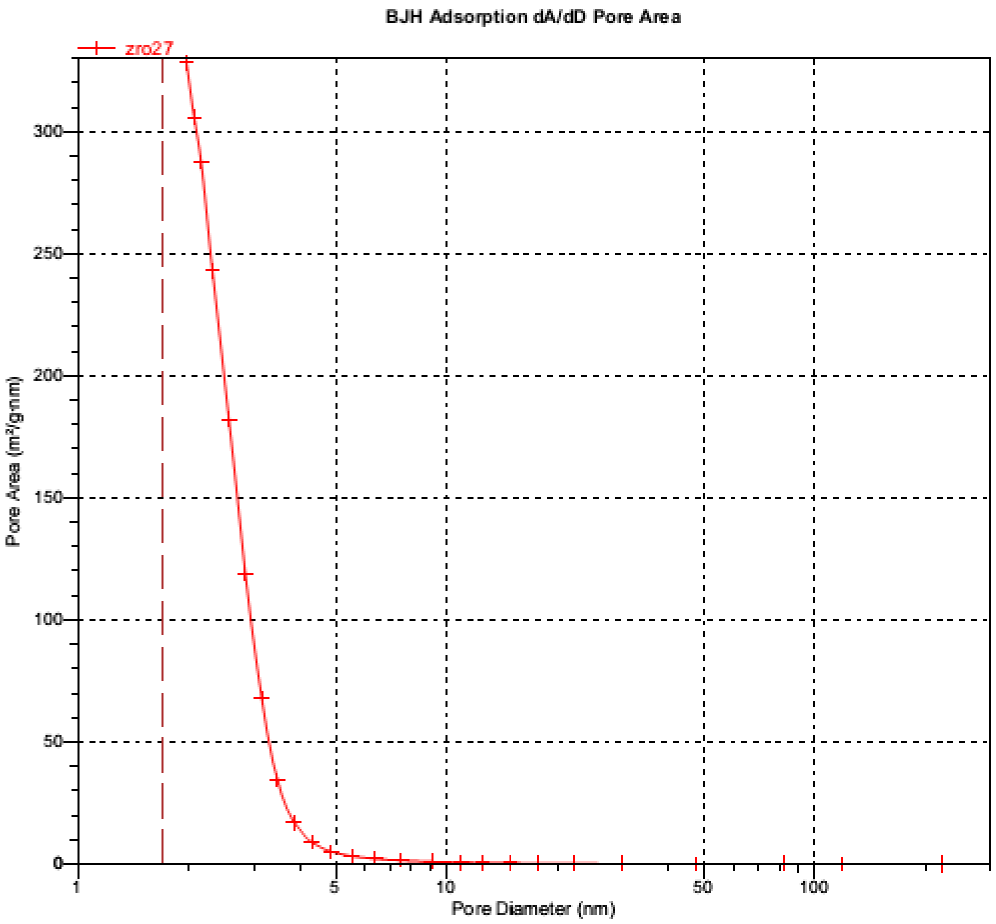

BJH Adsorption dA/dD Pore Area
zro27
Pore Area (m²/g·nm)
300
250
200
150
100
50
0
1
5
10
50
100
Pore Diameter (nm)

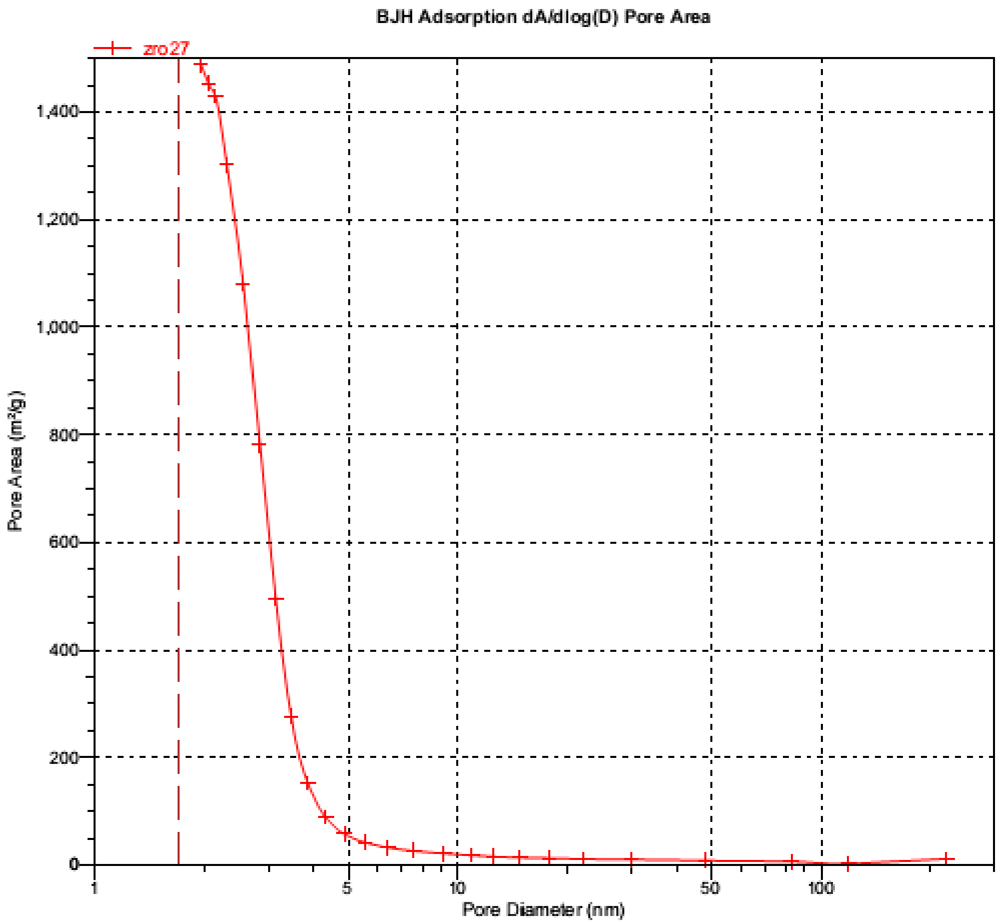

BJH Adsorption dA/dlog(D) Pore Area
zro27
Pore Area (m²/g)
1,400
1,200
1,000
800
600
400
200
0
1
5
10
50
100
Pore Diameter (nm)

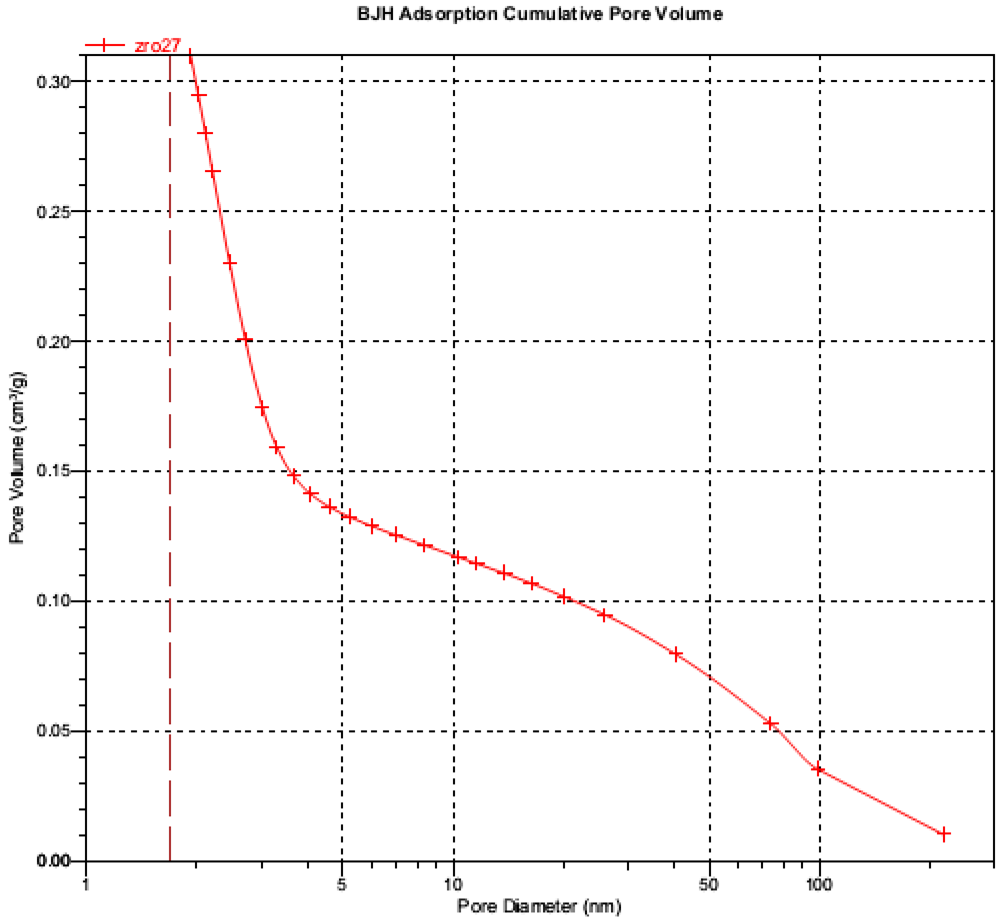

BJH Adsorption Cumulative Pore Volume
zro27
Pore Volume (cm³/g)
0.30
0.25
0.20
0.15
0.10
0.05
0.00
1
5
10
50
100
Pore Diameter (nm)

BJH Desorption Pore Distribution Report

t = 3.54 [-5 / ln(P/Po)] ^ 0.333

Diameter Range: 1.7000 nm to 300.0000 nm
Adsorbate Property Factor: 0.95300 nm
Density Conversion Factor: 0.0015468
Fraction of Pores Open at Both Ends: 0.00

Pore Diameter Range (nm)	Average Diameter (nm)	Incremental Pore Volume (cm³/g)	Cumulative Pore Volume (cm³/g)	Incremental Pore Area (m²/g)	Cumulative Pore Area (m²/g)
225.5 - 177.8	196.0	0.016037	0.016037	0.327	0.327
177.8 - 84.0	100.6	0.010193	0.026230	0.405	0.732
84.0 - 67.5	73.9	0.009365	0.035594	0.507	1.239
67.5 - 41.6	48.5	0.025980	0.061574	2.144	3.383
41.6 - 28.0	32.1	0.019862	0.081436	2.472	5.855
28.0 - 19.3	22.0	0.016239	0.097675	2.947	8.803
19.3 - 15.7	17.1	0.007118	0.104793	1.663	10.466
15.7 - 13.1	14.1	0.005435	0.110228	1.538	12.004
13.1 - 12.2	12.6	0.002016	0.112244	0.640	12.644
12.2 - 10.7	11.3	0.003276	0.115520	1.159	13.803
10.7 - 8.6	9.4	0.004954	0.120474	2.112	15.914
8.6 - 7.1	7.7	0.004252	0.124725	2.205	18.119
7.1 - 6.1	6.5	0.003625	0.128350	2.226	20.345
6.1 - 5.3	5.6	0.003413	0.131763	2.429	22.774
5.3 - 4.7	4.9	0.003236	0.134999	2.631	25.405
4.7 - 4.1	4.4	0.003280	0.138280	3.010	28.414
4.1 - 3.7	3.9	0.005721	0.144001	5.864	34.278
3.7 - 3.4	3.5	0.012572	0.156573	14.281	48.559
3.4 - 3.0	3.2	0.018966	0.175538	23.866	72.425
3.0 - 2.7	2.9	0.023533	0.199071	32.793	105.218
2.7 - 2.5	2.6	0.029021	0.228093	44.661	149.879
2.5 - 2.2	2.3	0.035220	0.263313	60.037	209.916
2.2 - 2.0	2.1	0.044453	0.307766	85.663	295.579

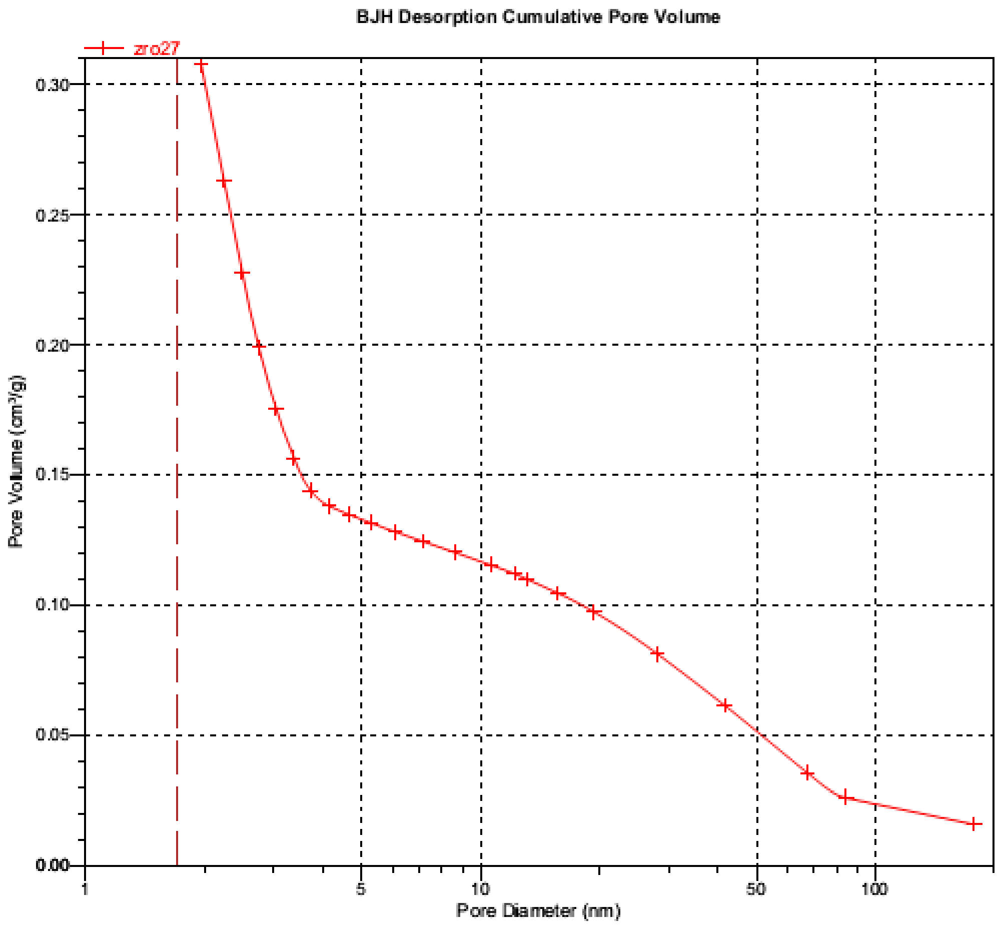
BJH Desorption Cumulative Pore Volume
zro27
0.30
0.25
0.20
0.15
0.10
0.05
0.00
Pore Volume (cm³/g)
1
5
10
50
100
Pore Diameter (nm)

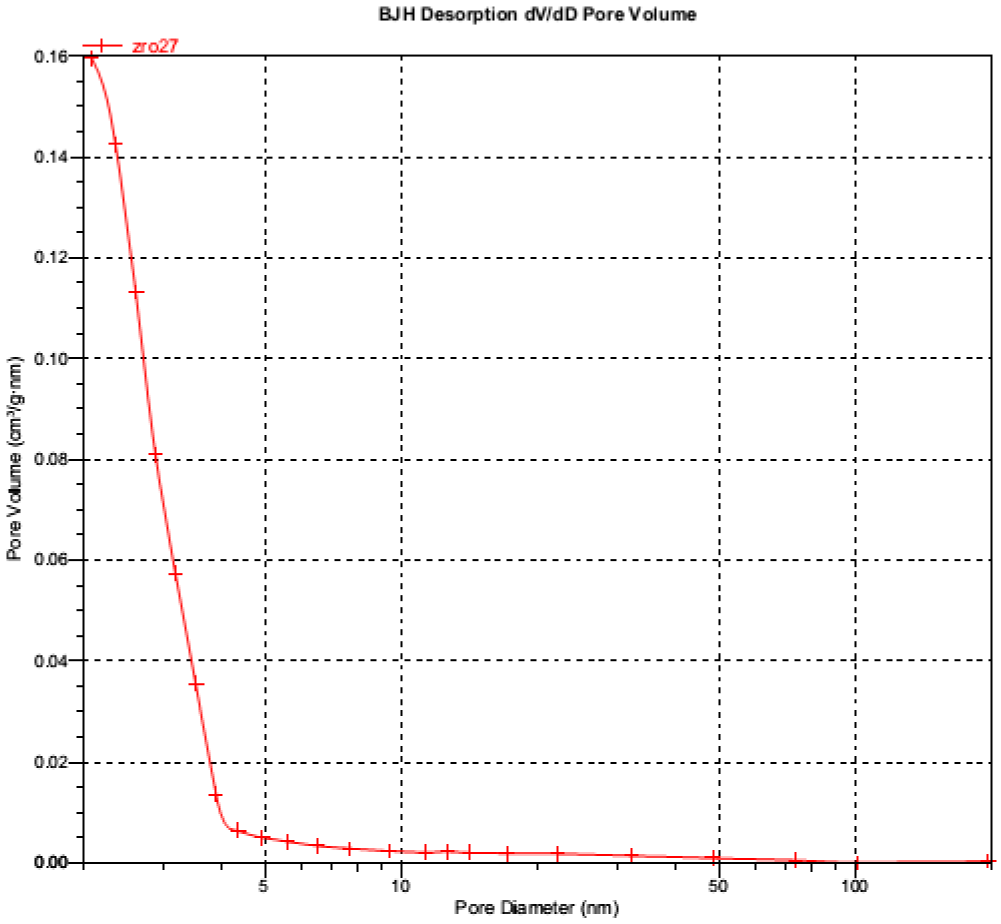

BJH Desorption dV/dD Pore Volume
zro27
Pore Volume (cm³/g·nm)
0.16
0.14
0.12
0.10
0.08
0.06
0.04
0.02
0.00
5
10
50
100
Pore Diameter (nm)

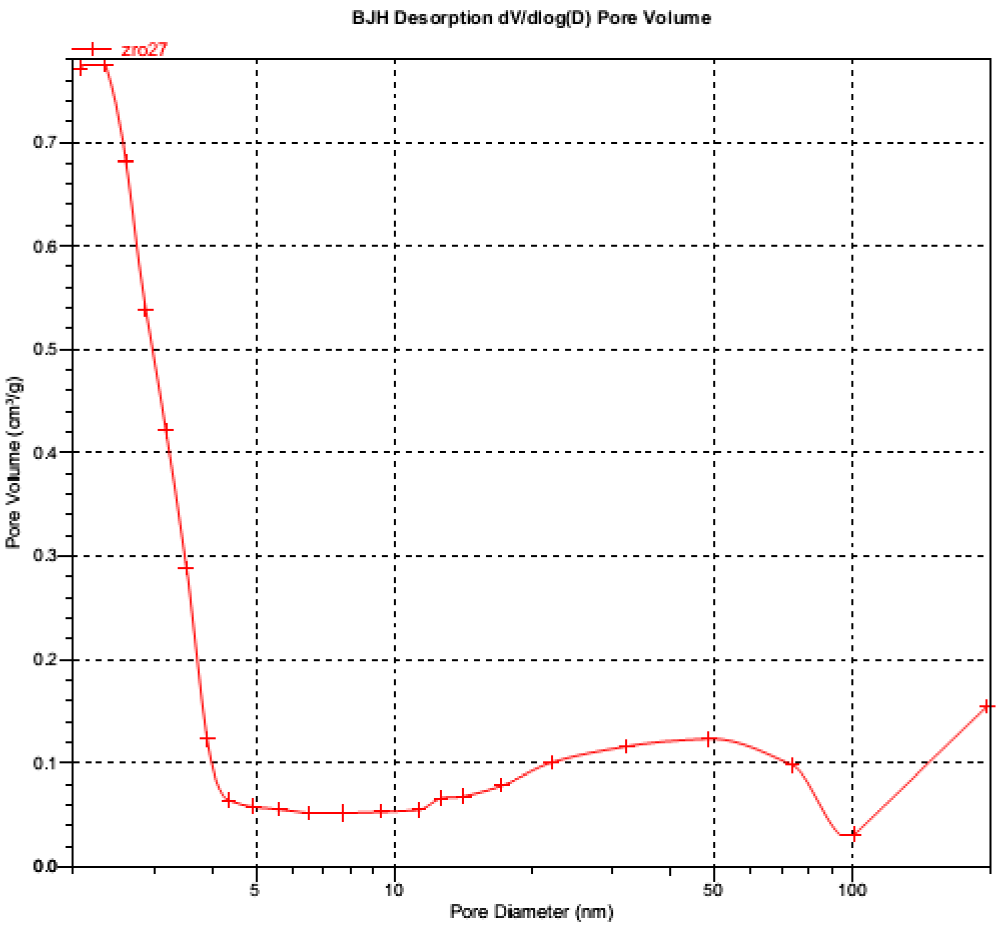

BJH Desorption dV/dlog(D) Pore Volume
zro27
Pore Volume (cm³/g)
0.0
0.1
0.2
0.3
0.4
0.5
0.6
0.7
5
10
50
100
Pore Diameter (nm)

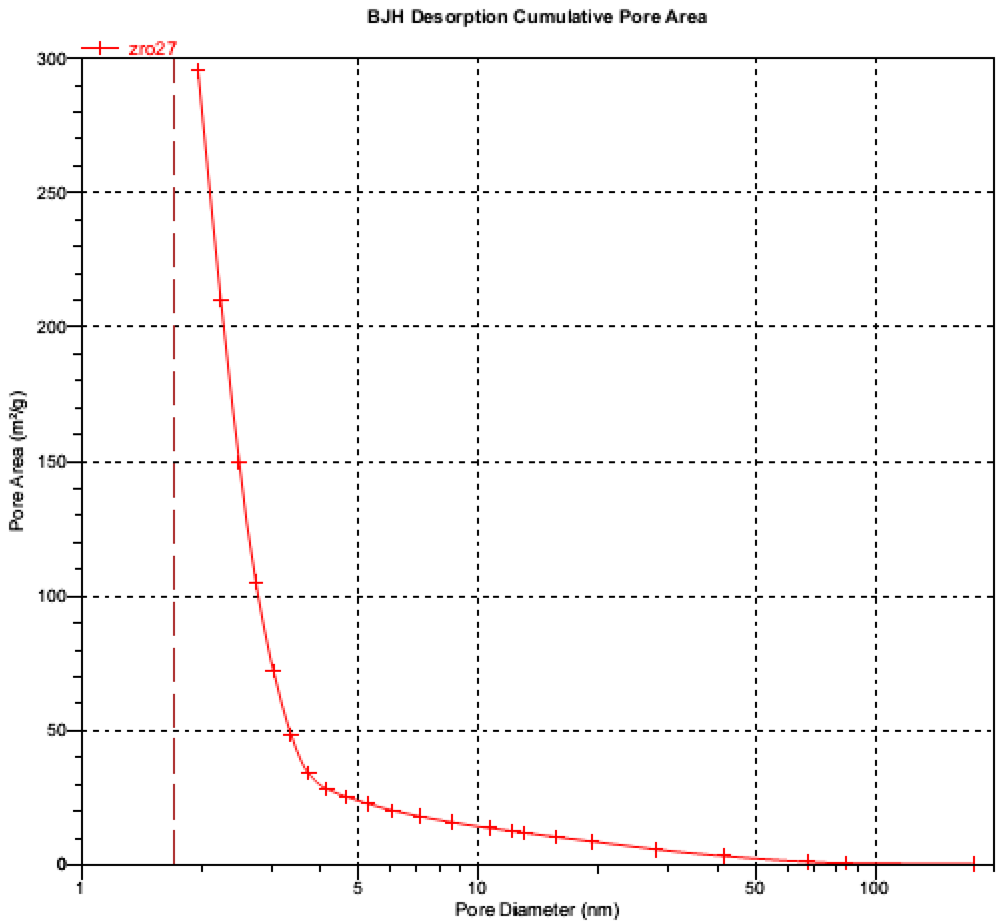
BJH Desorption Cumulative Pore Area
zro27
Pore Area (m²/g)
300
250
200
150
100
50
0
1
5
10
50
100
Pore Diameter (nm)

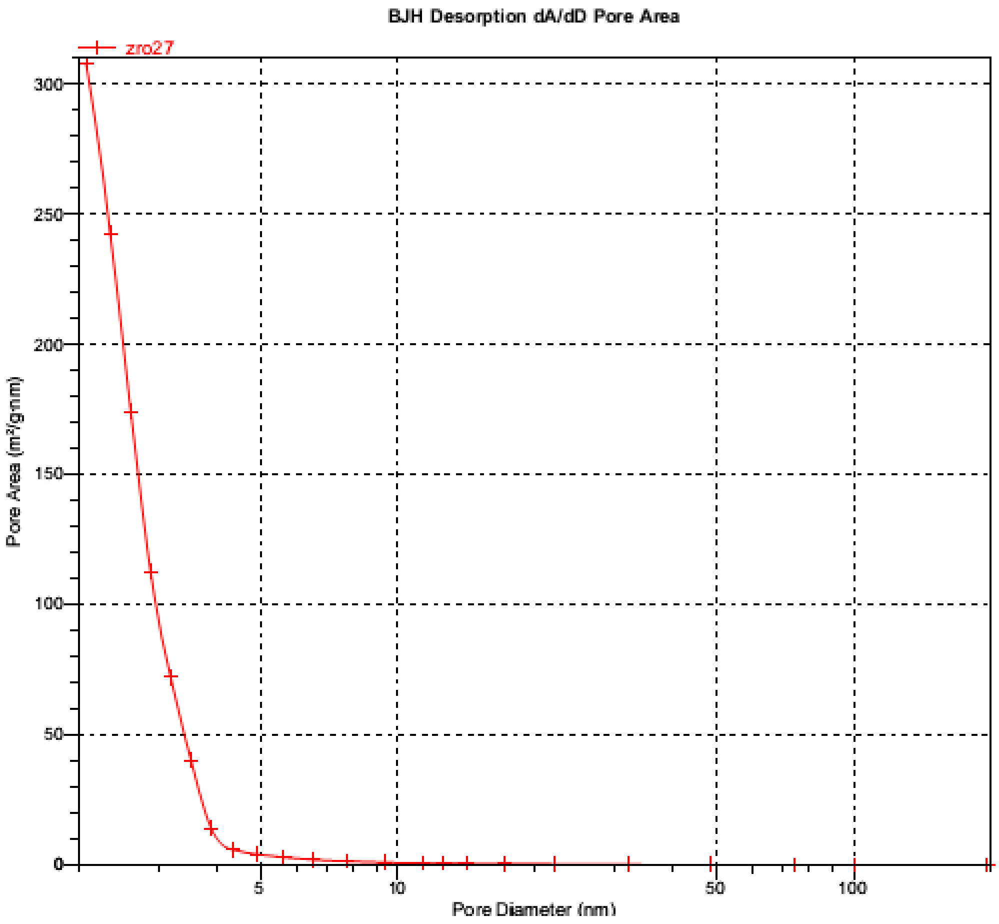

BJH Desorption dA/dD Pore Area
zro27
Pore Area (m²/g·nm)
300
250
200
150
100
50
0
5
10
50
100
Pore Diameter (nm)

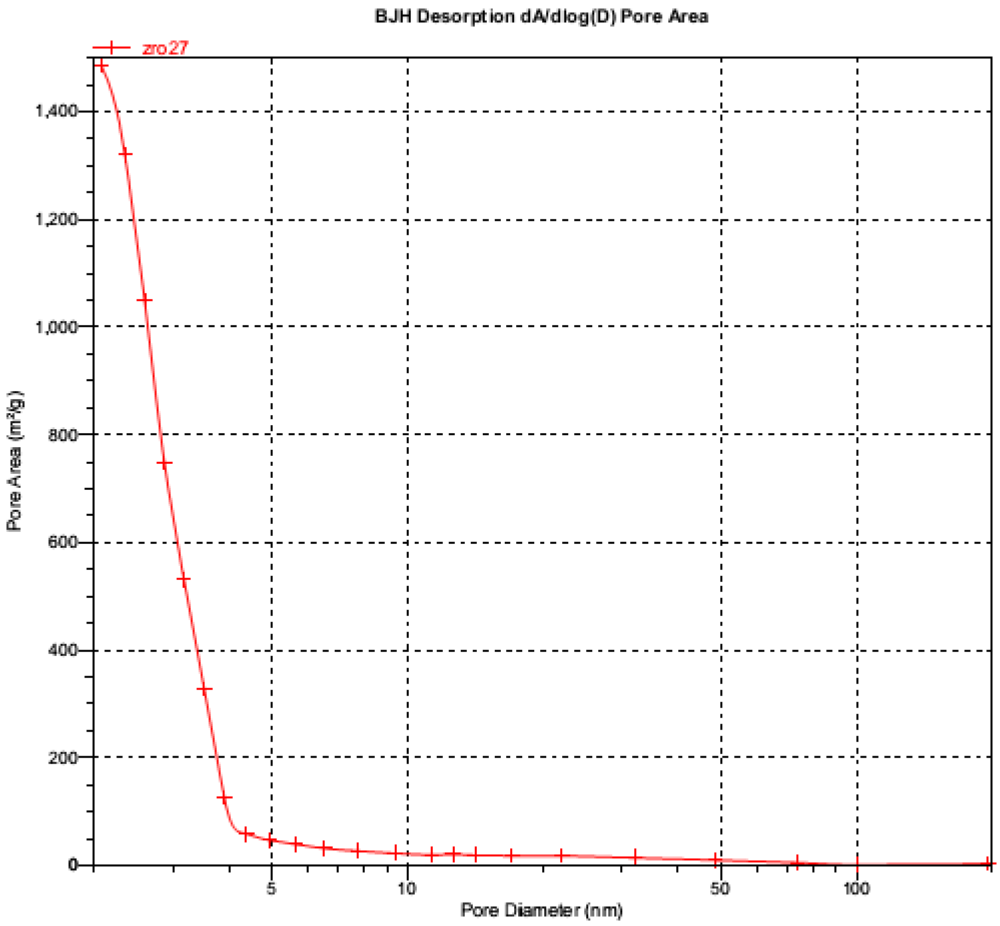

BJH Desorption dA/dlog(D) Pore Area
zro27
1,400
1,200
1,000
800
600
400
200
0
Pore Area (m²/g)
5
10
50
100
Pore Diameter (nm)

Summary Report

Surface Area

Single point surface area at P/Po = 0.197381660:	366.3377 m^2/g
BET Surface Area:	376.5479 m^2/g
Langmuir Surface Area:	485.0454 m^2/g
t-Plot External Surface Area:	415.0610 m^2/g
BJH Adsorption cumulative surface area of pores between 1.7000 nm and 300.0000 nm diameter:	297.8170 m^2/g
BJH Desorption cumulative surface area of pores between 1.7000 nm and 300.0000 nm diameter:	295.5793 m^2/g

Pore Volume

Single point adsorption total pore volume of pores less than 73.6426 nm diameter at P/Po = 0.972998912:	0.288718 cm^3/g
t-Plot micropore volume:	-0.019061 cm^3/g
BJH Adsorption cumulative volume of pores between 1.7000 nm and 300.0000 nm diameter:	0.309870 cm^3/g
BJH Desorption cumulative volume of pores between 1.7000 nm and 300.0000 nm diameter:	0.307766 cm^3/g

Pore Size

Adsorption average pore width (4V/A by BET):	3.06700 nm
BJH Adsorption average pore diameter (4V/A):	4.1619 nm
BJH Desorption average pore diameter (4V/A):	4.1649 nm

References

[1] http://www.zrchem.com/.

[2] http://us.mt.com/us/en/home.html.

[3] http://www.micromeritics.com/.

[4] http://www.vapourtec.co.uk/.

[5] http://www.knauer.net/en/downloads/pumps.html.

[6] http://thalesnano.com/h-cube.

[7] Raspberry Pi. http://www.raspberrypi.org (accessed December 16, 2013).
