# Supplementary figures and images for: Integration of enabling methods for the automated flow preparation of piperazine-2-carboxamide
Source: Beilstein J Org Chem. 2014 Mar 12;10:641–52. doi: 10.3762/bjoc.10.56 (PMC3999859; doi:10.3762/bjoc.10.56)

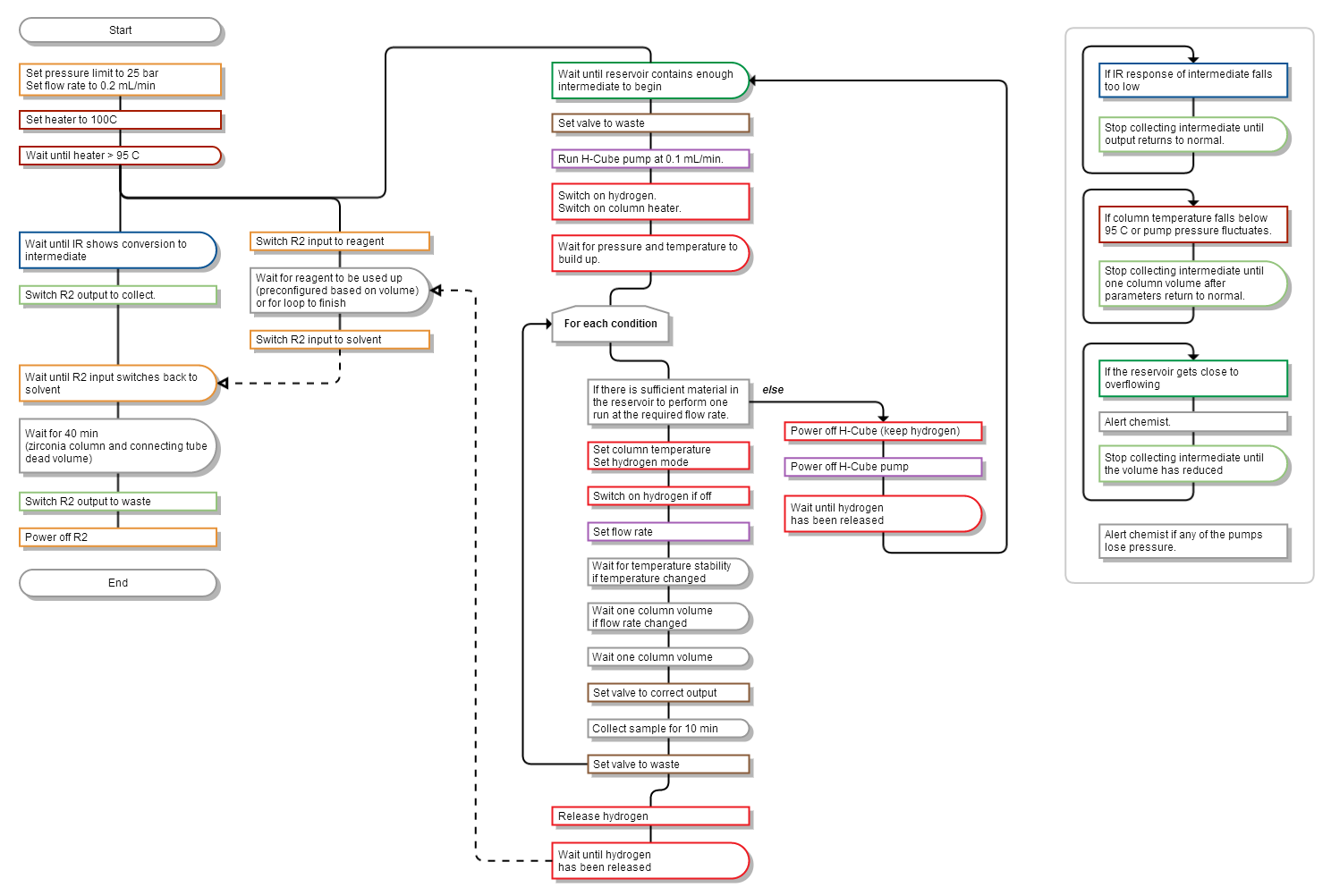

Supplement: File 5 — Flow chart representation of the control sequence for performing DoE experiments using intermediate from a reservoir. [file Beilstein_J_Org_Chem-10-641-s005.png]
